# Combined magnesium and silicon ions synergistically promote functional regeneration of skeletal muscle by regulating satellite cell fate

**DOI:** 10.1093/rb/rbaf008

**Published:** 2025-02-19

**Authors:** Hangbin Xia, Chen Yang, Huili Li, Lingwei Huang, Zhen Zeng, Runrun Chi, Ziwei Yang, Yuzen Wang, Jiang Chang, Yiren Jiao, Wenzhong Li

**Affiliations:** School of Ophthalmology and Optometry, School of Biomedical Engineering, Wenzhou Medical University, Wenzhou, Zhejiang 325027, China; Joint Centre of Translational Medicine, The First Affiliated Hospital of Wenzhou Medical University, Wenzhou 325000, China; National Engineering Research Center of Ophthalmology and Optometry, Eye Hospital Wenzhou Medical University, Wenzhou, Zhejiang 325027, China; National Clinical Research Center for Ocular Diseases, Eye Hospital, Wenzhou Medical University, Wenzhou, Zhejiang 325027, China; Joint Centre of Translational Medicine, The First Affiliated Hospital of Wenzhou Medical University, Wenzhou 325000, China; Zhejiang Engineering Research Center for Tissue Repair Materials, Wenzhou Institute, University of Chinese Academy of Sciences, Wenzhou 325000, China; School of Ophthalmology and Optometry, School of Biomedical Engineering, Wenzhou Medical University, Wenzhou, Zhejiang 325027, China; Joint Centre of Translational Medicine, The First Affiliated Hospital of Wenzhou Medical University, Wenzhou 325000, China; National Engineering Research Center of Ophthalmology and Optometry, Eye Hospital Wenzhou Medical University, Wenzhou, Zhejiang 325027, China; National Clinical Research Center for Ocular Diseases, Eye Hospital, Wenzhou Medical University, Wenzhou, Zhejiang 325027, China; Joint Centre of Translational Medicine, The First Affiliated Hospital of Wenzhou Medical University, Wenzhou 325000, China; Zhejiang Engineering Research Center for Tissue Repair Materials, Wenzhou Institute, University of Chinese Academy of Sciences, Wenzhou 325000, China; Joint Centre of Translational Medicine, The First Affiliated Hospital of Wenzhou Medical University, Wenzhou 325000, China; Zhejiang Engineering Research Center for Tissue Repair Materials, Wenzhou Institute, University of Chinese Academy of Sciences, Wenzhou 325000, China; School of Ophthalmology and Optometry, School of Biomedical Engineering, Wenzhou Medical University, Wenzhou, Zhejiang 325027, China; National Engineering Research Center of Ophthalmology and Optometry, Eye Hospital Wenzhou Medical University, Wenzhou, Zhejiang 325027, China; National Clinical Research Center for Ocular Diseases, Eye Hospital, Wenzhou Medical University, Wenzhou, Zhejiang 325027, China; School of Ophthalmology and Optometry, School of Biomedical Engineering, Wenzhou Medical University, Wenzhou, Zhejiang 325027, China; National Engineering Research Center of Ophthalmology and Optometry, Eye Hospital Wenzhou Medical University, Wenzhou, Zhejiang 325027, China; National Clinical Research Center for Ocular Diseases, Eye Hospital, Wenzhou Medical University, Wenzhou, Zhejiang 325027, China; School of Ophthalmology and Optometry, School of Biomedical Engineering, Wenzhou Medical University, Wenzhou, Zhejiang 325027, China; National Engineering Research Center of Ophthalmology and Optometry, Eye Hospital Wenzhou Medical University, Wenzhou, Zhejiang 325027, China; National Clinical Research Center for Ocular Diseases, Eye Hospital, Wenzhou Medical University, Wenzhou, Zhejiang 325027, China; Joint Centre of Translational Medicine, The First Affiliated Hospital of Wenzhou Medical University, Wenzhou 325000, China; Zhejiang Engineering Research Center for Tissue Repair Materials, Wenzhou Institute, University of Chinese Academy of Sciences, Wenzhou 325000, China; Joint Centre of Translational Medicine, The First Affiliated Hospital of Wenzhou Medical University, Wenzhou 325000, China; Zhejiang Engineering Research Center for Tissue Repair Materials, Wenzhou Institute, University of Chinese Academy of Sciences, Wenzhou 325000, China; School of Ophthalmology and Optometry, School of Biomedical Engineering, Wenzhou Medical University, Wenzhou, Zhejiang 325027, China; National Engineering Research Center of Ophthalmology and Optometry, Eye Hospital Wenzhou Medical University, Wenzhou, Zhejiang 325027, China; National Clinical Research Center for Ocular Diseases, Eye Hospital, Wenzhou Medical University, Wenzhou, Zhejiang 325027, China

**Keywords:** skeletal muscle satellite cells, silicon ions, magnesium ions, synergistic effect, muscle injury, Notch1 signal pathway

## Abstract

Muscle satellite cells (MuSCs) play a vital role in skeletal muscle regeneration. However, in intractable muscle diseases such as volumetric muscle loss (VML), the quantity and function of MuSCs are significantly reduced, severely limiting the body's inherent muscle regeneration capability. In this study, we propose a novel strategy to modulate the fate of MuSCs using a combination of bioactive magnesium (Mg) and silicon (Si) ions, sustainably delivered through magnesium silicate (MgSiO_3_, MS) bioceramic-based scaffolds. *In vitro*, Mg and Si ions synergistically promote the proliferation and differentiation of MuSCs. Similarly, Mg and Si ions derived from MS/poly(L-lactic acid) (MS/PLLA) composite scaffold also increase the proliferation and differentiation ability of MuSCs. Furthermore, MS/PLLA composite scaffolds facilitate the activation of MuSCs, regeneration of muscle fiber and neovascularization, while inhibiting fibrosis, thereby effectively restoring muscle function and promoting tibialis anterior muscle functional regeneration in a VML mouse model. Mechanistically, the combination of Mg and Si ions promotes the activation and proliferation of MuSCs by activating the Notch1-Hes1 pathway. Besides, the combination of Mg and Si ions also improves the differentiation of MuSCs by up-regulating Myod and Myog, and enhances fusion by up-regulating Mymk and Mymx expression. The outcomes of our research introduce a promising approach to the treatment of skeletal muscle injuries and related diseases.

## Graphical abstract

**Figure:**
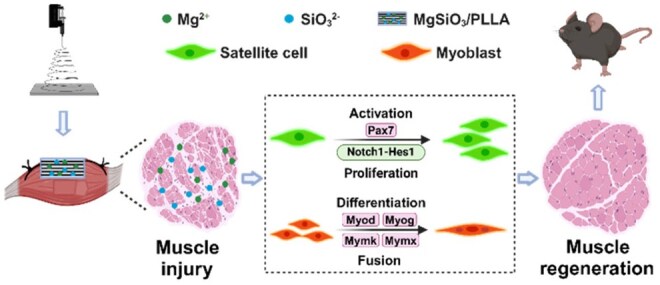

## Introduction

Skeletal muscles make up 30–40% of the average human body weight and play a fundamental role in maintaining voluntary movement and various physiological functions [1]. However, skeletal muscles are also one of the most easily injured tissues in the human body [2]. Sports injuries, traffic accidents, surgical procedures, etc. can all cause skeletal muscle damage. Most traumatic muscle injuries can be healed on their own or treated conservatively through graded exercise therapy and other plans [3]. In the process of skeletal muscle injury repair, muscle satellite cells (MuSCs) play a crucial role. MuSCs are adult myogenic stem cells located between the muscle fiber membrane and the substrate, usually in a resting state. When skeletal muscles are injured or induced, MuSCs are activated and begin to proliferate, then migrate to the damaged area, fuze with existing muscle fibers to promote muscle fiber regeneration and finally repair damaged muscles [4, 5]. However, for patients with muscle diseases or disorders, such as Duchenne muscular dystrophy or muscle atrophy, not only does the incidence of muscle injuries significantly increase, but the quantity and function of MuSCs are greatly reduced, seriously restricting the body's congenital muscle regeneration ability, making it difficult for exercise and other conservative treatment methods to achieve their treatment effects [6]. Similar phenomenon also occurs in patients with volumetric muscle loss (VML). Although the direct transplantation of exogenous MuSCs has supplemented the function of endogenous MuSCs to some extent, issues such as the source of exogenous MuSCs, *in vitro* proliferation rates, *in vivo* implantation efficiency and biosafety significantly limit its practical clinical application. Therefore, how to fully activate endogenous MuSCs in the body, and promote their proliferation, differentiation and fusion is still the key to exerting the muscle repair function of MuSCs and accelerating the regeneration of damaged muscle tissues.

Growth factors and small molecule compounds are the most common interventions for MuSCs. Studies have shown that local delivery of hepatocyte growth factors (HGFs) can activate skeletal MuSCs and increase myogenic cell numbers after injury [7]. Similarly, fibroblast growth factors (FGFs), such as FGF2 and FGF6, are critical for MuSCs self-renewal and promote proliferation and muscle regeneration by activating ERK/MAPK, p38α/β MAPKs and PI3K signaling pathways [8, 9]. In addition, small molecule inhibitors or agonists, like nicotinamide N-methyltransferase inhibitors (NNMTi), can activate aged muscle stem cells, enhancing their proliferation and fusion, thereby improving muscle regeneration and functional recovery in the elderly [10]. AdipoRon, an orally active small molecule agonist, alleviates oxidative stress and inflammation in Duchenne muscular dystrophy mice and promotes satellite cell differentiation and muscle regeneration via AMPK signaling [11]. However, the research on using growth factors or small molecules to regulate MuSCs for muscle repair is still in its early stages. The high cost and low stability of growth factors and the short half-life of small molecules limit their application in treating refractory muscle diseases [12].

Ion therapy strategies have emerged as a novel intervention method in recent years, proving effective in treating various tissue injuries and diseases. The core principle involves supplementing specific active ions to regulate key regenerative cells, thereby promoting tissue repair [13, 14]. Previous studies have demonstrated that magnesium (Mg) ions are essential for maintaining skeletal muscle integrity, and optimal Mg levels are essential for muscle health [15]. As the fourth most abundant cation in the human body, Mg is mostly stored in skeletal muscle, with cytoplasmic Mg concentrations around 0.8 mM. Mg deficiency has been shown to inhibit skeletal muscle growth and downregulate the expression of genes involved in skeletal muscle regeneration and energy metabolism [16, 17]. Myotubes cultured in a low extracellular Mg environment showed a significant reduction in thickness, downregulation of myosin heavy chain (MyHC), Myog and Myomixer (Mymx), and altered myotube metabolic profile [18]. Besides, in the skeletal muscle of Mg-deficient rats, mitochondria were swollen, the sarcoplasmic reticulum network was disrupted and there was a significant increase in the production of hydroxyl free radicals in the muscle homogenate [19]. Instead, dietary Mg helps preserve age-related losses in skeletal muscle mass and strength in women of all ages [20]. Also, Mg supplementation can effectively mitigate corticosteroid-related muscle atrophy in rats [21] and prevent skeletal muscle injuries in athletes [22]. However, no reports have been found on using Mg ions to treat refractory muscle injuries. Liu *et al.* reported that Mg supplementation only promotes myogenic cell differentiation without affecting their activity and proliferation, suggesting that Mg ions alone have limited effects on MuSCs regulation and skeletal muscle regeneration [23]. In contrast, silicon (Si) ions have shown potential in promoting C2C12 muscle cell proliferation and differentiation, thereby aiding muscle regeneration [24–26]. Our previous studies also indicate that Si ions can enhance the biological effects of active metal ions, synergistically promoting tissue repair [27–29]. We hypothesized that a combination of Mg and Si ions could synergistically activate skeletal MuSCs, enhancing their proliferation, differentiation and fusion, thereby accelerating regeneration and functional recovery following skeletal muscle injury.

On the other hand, in practical applications, considering the mode of intervention is as important as focusing on the core functional components. For muscle regeneration, material-based interventions, particularly those with muscle-oriented structures, can provide robust growth environments and alignment guidance for muscle stem cells, thereby accelerating the regeneration process. Poly(L-lactic acid) (PLLA), an FDA-approved material with excellent biocompatibility, mechanical properties and ease of processing, has been widely utilized in tissue engineering, including applications in muscle regeneration [30–32]. For instance, oriented PLLA scaffolds prepared through electrospinning technology have been shown to support the formation of muscle tubes with aligned myoblasts, promoting the assembly of striated muscle fibers and enhancing skeletal muscle regeneration [33, 34]. These results suggest that electrospun PLLA-based materials may serve as promising candidates for studying the regulation of MuSCs. To better validate our hypothesis, this study begins by exploring the synergistic effects of Mg and Si ions on the regulation of MuSCs. Subsequently, we prepared magnesium silicate (MgSiO_3_, MS) bioceramics capable of co-releasing Mg and Si ions, which were then combined with PLLA by electrospinning technology to create MS/PLLA composite scaffolds for defect-filling. Finally, we constructed a mouse model with large-scale muscle defects to investigate the *in vivo* repair effects on skeletal muscle injuries. Additionally, cellular experiments were conducted to analyse the molecular biological mechanisms by which the combination of Mg and Si ions promotes skeletal muscle regeneration. This study aims to provide new therapeutic strategies and theoretical foundations for muscle injury treatment.

## Materials and methods

### Isolation and purification of MuSCs

Eight-week-old male C57BL/6 mice were purchased from the Zhejiang Provincial Experimental Animal Center. The National Institutes of Health's guidelines for animal care and use were strictly followed for all animal experiments, which were also approved by the Experimental Animal Ethics Committee of the Wenzhou Research Institute, Chinese Academy of Sciences (Protocol No. WIUCAS23032902). To isolate MuSCs, mice were first anesthetized, and the hindlimb muscles were subsequently dissected under sterile conditions. The muscles were rinsed with PBS and cut into 1 mm^3^ pieces. The muscle pieces were incubated in 0.2% collagenase II at 37°C for 1 h, shaken every 10 min. After that, a growth medium was added to terminate the digestion, and the cell suspension was filtered through 100, 200 and 400-mesh cell strainers. After centrifuging the filtered cell suspension, the cells were resuspend and transferred to T25 flasks. The cells were then cultured for 2 h before being purified using differential adhesion. The non-adherent cells are primarily satellite cells, which can be transferred to new culture flasks for further culture. The P2-P3 MuSCs were used for subsequent experiments.

MuSCs (1.0 × 10^4^ cells/well) were seeded in 48-well plates and cultured for 24 h. The cells were then fixed with 4% paraformaldehyde and permeabilized with 0.5% Triton X-100. After blocking with 5% bovine serum albumin (BSA), the cells were incubated overnight at 4°C with diluted Pax7 antibody. Following incubation with secondary antibodies for 2 h at room temperature, the cell nuclei were stained with DAPI. Pax7-positive cells were visualized and imaged using an inverted fluorescence microscope (Axio Vert.A1, ZEISS, Germany).

### Comparison of the effects of Mg, Si and their combination on the proliferation and differentiation of MuSCs

For concentration screening experiments, MuSCs were seeded in a 96-well plate and cultured in growth medium supplemented with varying concentrations of MgCl_2_ (1.25, 2.5, 5, 10 and 20 mM) (M116331, Aladdin, China) and Na_2_SiO_3_ (0.05, 0.1, 0.5, 1 and 2 mM) (S108360, Aladdin, China) for 3 days. Subsequently, the cell viability of MuSCs was assessed according to the instruction of Cell Counting Kit-8 (CCK-8) (40203ES76, Yeasen Biotechnology, China). Absorbance at 450 nm was measured using a microplate reader (EPOCH2NS, BioTek Instruments, USA).

The MuSCs (1.0 × 10^4^ cells/well) were seeded in 48-well plates and cultured for 48 h. Subsequently, the EdU solution was diluted 1:1000 with a complete medium and added to the cell culture plate for another 2 h. The cells were then fixed with 4% paraformaldehyde and permeabilized with 0.5% TritonX-100. After that, the cells were stained according to the instructions provided with the EdU staining kit (R11053.9, Ruibo, China). The EdU-positive MuSCs were visualized and captured using an inverted fluorescence microscope (Axio Vert.A1, ZEISS, Germany). The percentage of positive cells was then analysed using Image-J.

The MuSCs (1.0 × 10^4^ cells/well) were seeded in 48-well plates and cultured for 48 h. When the cells reached 100% confluence, the growth medium was replaced with differentiation medium, and the cells were cultured for an additional 5 days before fixation and permeabilization. After blocking with 5% BSA at room temperature for 2 h, the cells were incubated overnight at 4°C with diluted Myod and Myosin antibodies. Following incubation with secondary antibodies for 2 h at room temperature, DAPI was used to stain the cell nuclei. The Myod- and Myosin-positive MuSCs were visualized and imaged using an inverted fluorescence microscope (Axio Vert.A1, ZEISS, Germany). The percentage of positive cells was then quantified using Image-J.

### Preparation of MS and MS/PLLA composite scaffolds

The sol–gel method was used to synthesize MS powder. The specific experimental steps are as follows: First, add an appropriate amount of dilute HNO_3_ (2 mol/l) to deionized water, then measure 0.2 mol of tetraethyl orthosilicate (TEOS) and slowly add it to the above-mentioned deionized water under stirring at room temperature. After the addition of TEOS is completed, stir until it is clear and transparent to obtain silica sol. Then, according to the stoichiometric ratio (Mg:Si = 1:1), weigh 0.2 mol of Mg(NO_3_)_2_·6H_2_O into the above-mentioned sol and continue stirring at room temperature until a uniform and transparent solution is formed. The obtained transparent sol is sealed and then aged at 60°C for 24 h to form a transparent wet gel. Then, the obtained wet gel is unsealed and dried at 120°C for 48 h to obtain a completely dried dry gel. After grinding and sieving, the dry gel is calcined at 1200°C for 3 h. Finally, the calcined powder is ball-milled and sieved using a planetary ball mill to obtain MS powder.

PLLA scaffolds containing different proportions of MS (HQ-MS, Kunshan Chinese Technology New Materials, China) (0%, 2%, 4% and 8%) were prepared using electrospinning. First, 0, 0.02, 0.04 and 0.08 g of MS were added to 10 ml of hexafluoroisopropanol (HFIP) (920-66-1, Aladdin, China), respectively, and ultrasonically dispersed for 30 min. Then, the suspensions were continuously stirred on a magnetic stirrer. Next, 1 g of PLLA (Jinan Daigang, Biomaterial, China) was added to each suspension and stirred overnight to obtain a homogeneous spinning solution. The spinning solutions were added to 10 ml syringes and connected to the cathode of a high-voltage electrospinning machine (TL-01, Shenzhen Tongli Micro-Nano Technology Co., Ltd) for electrospinning. The prepared composite scaffolds were vacuum-dried for 24 h to completely remove residual solvents before further characterization. The composite scaffolds with MS in different proportions were named PLLA, 2%MS/PLLA, 4%MS/PLLA and 8%MS/PLLA, respectively.

### Characterization of MS and MS/PLLA composite scaffolds

The crystalline phase of MS powder, PLLA and MS/PLLA scaffolds was analysed by X-ray diffractometer (XRD, Bruker AXS D8). Fourier transform infrared spectroscopy (FTIR, Bruker Tensor II) was used to analyse the molecular structure, functional groups and chemical composition of MS powder, PLLA and MS/PLLA scaffolds. The morphology and elemental distribution of MS powder, PLLA and MS/PLLA were analysed by field emission scanning electron microscope (SEM, SU-8010, Japan) equipped with an energy dispersive spectrometer (EDS). The orientation degree of electrospinning membranes was also statistically analysed by Image-J.

To measure the release of Mg and Si ions from MS/PLLA composite scaffolds, composite scaffolds (250 mg) with different weight ratios of MS (2%, 4% and 8%) were immersed in 2 ml of Tris-HCl (pH = 7.4) solution at 37°C and 160 rpm/min. The release solution was collected after soaking for 1, 3, 7 and 14 days. The concentrations of Mg and Si ions were analysed by inductively coupled plasma mass spectrometry (ICP-MS, Agilent 7850).

An accelerated degradation testing method was employed to determine the degradation profile of the different composite scaffolds. Briefly, samples were immersed in 15 ml of degradation solution (0.01 M NaOH) and incubated in a shaker at 37°C and 160 rpm. At specific time points (day 1, day 3, day 7 and day 14), samples were removed, washed with deionized water and dried in a vacuum oven at 60°C. The mass of the scaffolds before and after degradation was measured using an electronic balance. In addition, the degradation profile of PLLA and 4%MS/PLLA scaffolds in simulated body fluid (SBF) was conducted. Briefly, samples were immersed in 15 ml of SBFs and incubated in a shaker at 37°C and 160 rpm. At specific time points (day 1, day 3, day 7, day 14 and day 28), samples were removed, washed with deionized water and dried in a vacuum oven at 60°C. The mass of the scaffolds before and after degradation was measured using an electronic balance.

### Biocompatibility of MS/PLLA composite scaffold

For the biocompatibility experiment of MS/PLLA composite scaffold, the PLLA scaffolds composited with varying concentrations of MS were cut into 1.5 × 1.5 cm pieces, and spread on the bottom of a 48-well plate after disinfection and sterilization. Then, MuSCs were seeded at 1.0 × 10^4^ cells/well onto PLLA or MS/PLLA composite scaffolds in 48-well plates and cultured for 48 h. Cell viability was assessed using the CCK-8 assay, following the manufacturer’s instructions. Briefly, 1/10 volume of CCK-8 solution was added to each well, and the plate was incubated for 2 h. Absorbance at 450 nm was measured using a microplate reader (EPOCH2NS, BioTek instruments, USA) to assess cell viability.

The survival of MuSCs was assessed with Live/Dead Cell Imaging Kit (R37601, Invitrogen, USA) following the manufactures’ instruction. Briefly, MuSCs were seeded at 1.0 × 10^4^ cells/well onto PLLA or MS/PLLA composite scaffolds in 48-well plates and cultured for 48 h. Component A (live green) was transferred into component B (dead red) to produce the working solution. Then, equal volume of working solution was added into each well and incubated at 25°C in dark for 15 min. The fluorescence images were captured by inverted fluorescence microscope (Axio observer A1, Zeiss, Germany). The live and dead MuSCs were investigated and imaged using an inverted fluorescence microscope (Axio Vert.A1, ZEISS, Germany).

### EdU assay

The MuSCs (1.0 × 10^4^ cells/well) were seeded onto PLLA or 4%MS/PLLA scaffolds in 48-well plates and cultured for 48 h. MuSCs were subjected to EdU detection following the same procedure outlined in Section ‘Preparation of MS and MS/PLLA composite scaffolds’ for MuSCs proliferation assay. Subsequently, the EdU solution was diluted 1:1000 with a complete medium and added to the cell culture plate for another 2 h. The cells were then fixed with 4% paraformaldehyde and permeabilized with 0.5% TritonX-100. After that, the cells were stained according to the instructions provided with the EdU staining kit (R11053.9, Ruibo, China). The EdU-positive MuSCs were visualized and captured using an inverted fluorescence microscope (Axio Vert.A1, ZEISS, Germany). The percentage of positive cells was then analysed using Image-J.

### Immunofluorescence assay of MuSCs

The MuSCs (1.0 × 10^4^ cells/well) were seeded onto PLLA or PLLA/4%MS electrospun membranes in 48-well plates and cultured for 48 h. Cell samples were sequentially fixed, permeabilized and blocked. Primary antibodies against Myod (sc-377460, Santa Cruz, USA), Myogenin (bs-3550R, Bioss, China), Notch1(bs-1335R, Bioss, China) and Hes1(bs-2972R, Bioss, China) were diluted and incubated separately. Secondary antibodies were then incubated. DAPI was used to stain the cell nuclei. The cells were visualized and captured. The percentage of positive cells was analysed using Image-J.

### Construction of VML mouse model and electrospinning membrane treatment

Thirty-two eight-week-old male C57BL/6 mice were purchased from the Zhejiang Provincial Experimental Animal Center and randomly divided into four groups, with eight mice in each group. Briefly, mice were anesthetized with a 1% sodium pentobarbital solution (1 ml/100 g). After general anesthesia, the tibialis anterior (TA) muscle was exposed through a skin incision. A VML model was created by excising a 3 × 3 × 6 mm longitudinal defect using fine iris scissors. The sterilized PLLA or 4%MS/PLLA scaffold was then placed over the defect, with both ends of the scaffold secured to the edges of the TA defect site using absorbable sutures. Finally, the skin was sutured closed. The groups were as follows: Sham group (no surgery was performed); VML group (VML model without any treatment at the injury site); VML + PLLA group (VML model and PLLA were implanted at the injury site); VML + MS/PLLA group (VML model and MS/PLLA were implanted at the injury site). After 28 days of treatment, TA muscles from each group were harvested from the mice for subsequent assays. The animal ethical approval of this study was approved by the Animal Research and Ethics Committee of Wenzhou Institute of University of Chinese Academy of Sciences (Approval Issue No. WIUCAS23032902).

### Hind limb grip force assay and fatigue testing

The hind limb grip strength of mice in each group was measured using a grip strength meter (LJ800-012, Nscing Es, China), following a previously described method [35]. A rotarod test was performed to assess the fatigue resistance of C57BL/6 mice. The mice were placed on a rotating rod, and the following parameters were set: initial speed, first speed, second speed, acceleration time and duration. The latency to fall from the rod was recorded and analysed.

### Histological staining and analysis

The TA muscles were collected, dehydrated, paraffin-embedded and sectioned into 5-µm-thick slices. After that, the paraffin sections were dewaxed, cleared and dehydrated using graded ethanol. Hematoxylin and eosin (H&E) staining (C0105S, Beyotime, China) and Masson’s trichrome staining (G1340, Solarbio, China) were then performed following the manufacturer's instructions. The stained sections were imaged with a bright-field microscope and analysed using Image-J (National Institutes of Health, USA). Collagen deposition was quantified as the collagen volume fraction, calculated as collagen area/total muscle area × 100%.

### Immunofluorescence staining of tissue sections

Muscle tissue samples were collected 28 days post-treatment. After fixation and dehydration, the samples were embedded in OCT and frozen sections were cut at 5 μm thickness. The frozen sections were then stained for Pax7, CD31, CD86 and CD206 by immunofluorescence. Briefly, the OCT was removed and the sections were permeabilized and blocked. Primary antibodies against Pax7 (sc-81648, Santa Cruz, USA), Laminin (L9393, Sigma, USA), CD31 (GB11063-2-100, Servicebio, China) were diluted and incubated overnight at 4°C. Secondary antibodies were diluted and incubated in the dark. The sections were then mounted with a DAPI-containing mounting medium and captured. The percentage of positive cells was analysed using Image-J.

### RNA isolation and real-time quantitative PCR analysis

Total RNA was isolated from mouse TA and MuSCs by using RNAiso Plus (9019, Takara, Japan), and was reverse transcribed into cDNA. Gene expression was quantified using a qPCR Master Mix Kit (Q311-03, Vazyme, China). Primer sequences are provided in Supplementary Table S1. Relative gene expression was calculated using 2^−ΔΔ^^*Ct*^ method.

### Western blot assay

Protein blot analysis was performed as previously described [36]. Muscle tissue samples were homogenized and lysed in RIPA buffer (P0013C, Beyotime, China) containing a protease inhibitor cocktail (P1005, Beyotime, China). The protein lysates were separated and then transferred to PVDF membranes using the Mini-PROTEAN Tetra Cell (1658004EDU, Bio-Rad, USA). The membranes were blocked with 5% skim milk buffer and then incubated with the primary antibody overnight at 4°C. The membranes were then incubated with HRP-conjugated secondary antibody for 2 h. Finally, protein bands were detected and imaged using enhanced chemiluminescence (FD8030, ECL, FDbio Science) with the ChemiDoc Imaging System (12003153, Bio-Rad, USA). Band intensities were quantified using Image-J and normalized to the band intensity of Gapdh. The antibodies used in this study are listed in Supplementary Table S2.

### Statistical analysis

Statistical analyses were conducted using a Prism software (Version 9.5, GraphPad, USA). The data were analysed using Student's *t*-tests or one-way analysis of variance (ANOVA) with Fisher's least significant difference (LSD) tests. Data are presented as mean ± standard error of the mean (SEM), and comparisons with *P* < 0.05 were considered statistically significant.

## Results

### Mg and Si ions synergistically promote the proliferation and differentiation of MuSCs

**Figure 1.**
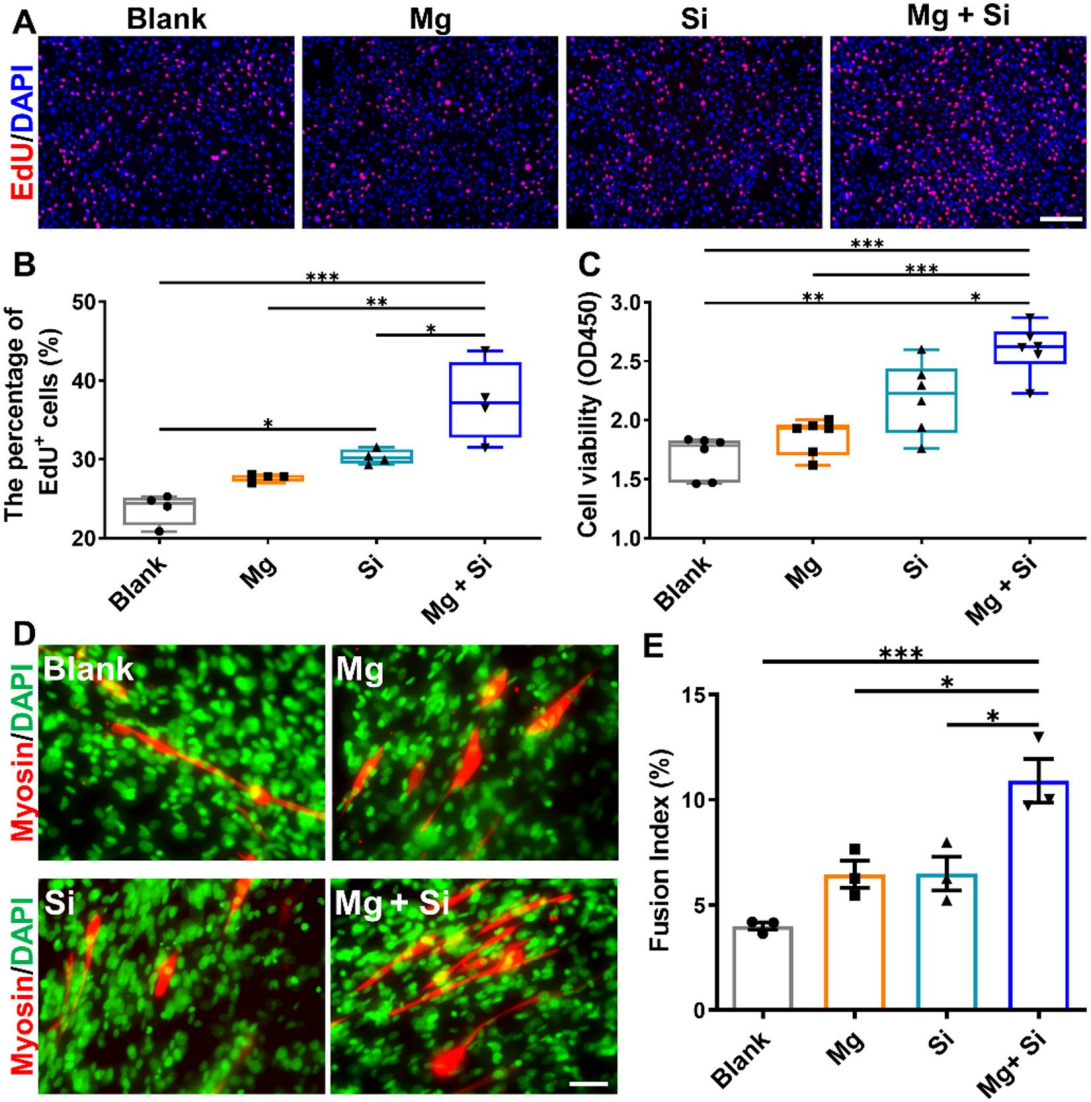
Synergistic effect of Mg and Si ions on the MuSCs. (**A**) EdU staining results of MuSCs, scale bar: 200 μm. (**B**) The percentage of EdU-positive MuSCs in each group. (**C**) The cell viability of MuSCs in each group after 48 h of different treatments. (**D**) Immunofluorescence staining of Myosin in MuSCs after 5 days of differentiation, scale bar: 50 μm. (**E**) Statistical results of the average fluorescence density of Myosin in each visual field of MuSCs in different groups. *n* = 3. *P < 0.05; **P < 0.01; ***P < 0.001.

Initially, MuSCs were isolated through meticulous mechanical and enzymatic digestion, as previously described [37], with their identity confirmed via Pax7 immunofluorescence staining. The results revealed a predominantly Pax7-positive population (Supplementary Figure S1), indicating that the collected cells are primarily skeletal MuSCs. Following induction with differentiation medium for 3 days, all MuSCs expressed the myogenic factor Myod (Supplementary Figure S2), which showed that skeletal MuSCs can differentiate into myoblasts. To explore the effects of Mg and Si ions on MuSCs, we conducted a screening for optimal concentrations. CCK-8 assay results revealed that varying concentrations of Mg ions had no discernible impact on MuSCs viability (Supplementary Figure S3A). However, the addition of 5 mM Mg increased the number of Myosin-positive myotubes (Supplementary Figure S3B and C). Similarly, the CCK-8 assay results revealed that 1 and 2 mM Si can significantly increase the viability of MuSCs (Supplementary Figure S4A). However, incorporating various concentrations of Si does not exert a notable impact on the differentiation of MuSCs (Supplementary Figures S4B and C). What is interesting, comparing with blank, single Mg, single Si, concurrent supplementation of Mg and Si significantly elevated proliferation (Figure 1A and B) and cell viability of MuSCs (Figure 1C). Additionally, the simultaneous introduction of Mg and Si further facilitated the differentiation and maturation of MuSCs (Figure 1D and E). In summary, the combined supplementation of Mg and Si ions synergistically enhances both the proliferation and differentiation of MuSCs *in vitro*.

### Characterization of MS/PLLA composite scaffolds

**Figure 2.**
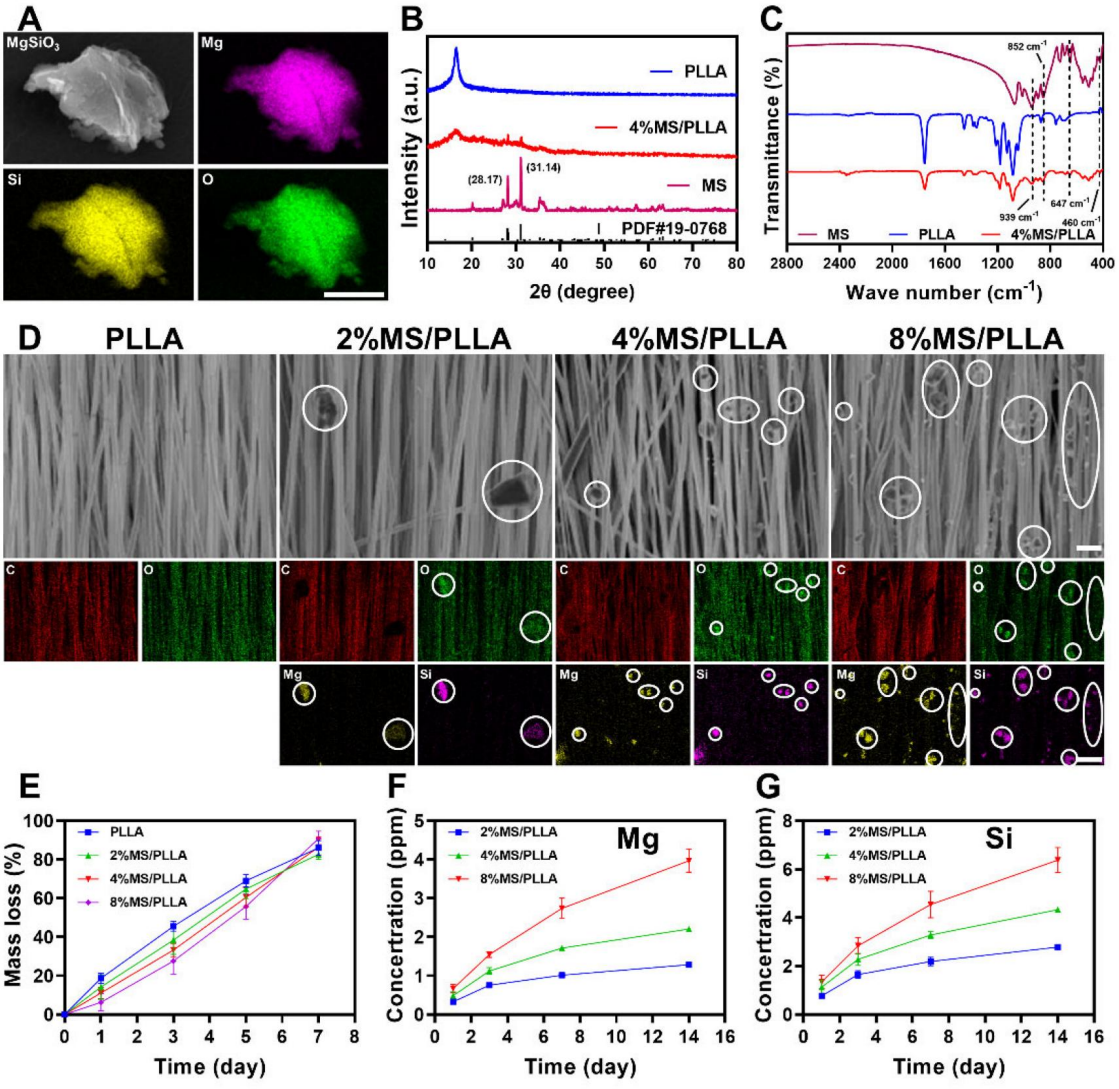
Characterization of MS/PLLA composite scaffolds. (**A**) The morphology and element mapping of MS powder by SEM and EDS. Scale bar: 2.5 μm. (**B**) The XRD spectrum of MS, PLLA and 4%MS/PLLA scaffolds. (**C**) The FTIR spectra results of MS, PLLA and 4%MS/PLLA scaffolds. (**D**) The morphology and elemental distribution of different scaffolds. Scale bar: 5 μm in SEM images and 10 μm in EDS images. The white circles represent MS. (**E**) The degradation curve of the prepared MS/PLLA composite scaffolds. The release curve of Mg (**F**) and Si (**G**) ions in the prepared MS/PLLA composite scaffolds at various time points.

To achieve the simultaneous release of Mg and Si ions, we prepared L-polylactic acid (PLLA) scaffolds compounded with MS (MS/PLLA). Scanning electron microscopy (SEM) images showed that MS particles had a size around several tens of microns, while the element mapping results indicated a uniform distribution of Mg, Si and O elements in the MS powder (Figure 2A). The XRD assay results further confirmed the crystal structure of MgSiO_3_, with the characteristic diffraction peaks of MS consistent with PDF #19-0768. Compared with PLLA scaffold, MS/PLLA scaffold exhibited two new diffraction peaks at 2*θ* = 28.17° and 31.14°, which correspond to the diffraction peaks of MS, suggesting the success composition of MS in PLLA (Figure 2B). The FTIR spectra of PLLA before and after MS composite show that the functional groups have changed significantly. The peak at 939, 852 and 647 cm^−1^ corresponds to the bending vibration of Si–O–Si in the silicon–oxygen tetrahedron, while the peak at 460 cm^−1^ corresponds to the vibration of the Mg–O bond (Figure 2C). The SEM results showed that as the concentration of composite MS increased, the number of MS particles within the scaffold gaps or wrapped around the scaffold also increased significantly. EDS analysis revealed that, with higher composite concentrations of MS, the levels of Mg and Si elements in the scaffold gradually increased (Figure 2D). These findings suggest that MS was successfully incorporated into the PLLA scaffold and distributed evenly throughout. Compared to the PLLA group, compounding different concentrations of MS did not affect the orientation of the scaffold (Supplementary Figure S5). However, the tensile strength of the MS/PLLA composite scaffolds improved with the increase of MS (Supplementary Figure S6), with mechanical properties closely resembling the tensile strength of mouse skeletal muscle.

To better understand the degradation and ion release behavior of MS/PLLA scaffolds. We conducted several experiments. The *in vitro* accelerated degradation testing in alkaline conditions showed that all the MS/PLLA groups could gradually degrade, and the composite scaffolds with higher content of MS degraded slightly faster with no significant differences (Figure 2E). Subsequently, we incubated the PLLA and 4%MS/PLLA scaffolds in SBF to better simulate the *in vivo* degradation process. The results showed that the degradation rate of the PLLA scaffold was slightly higher than that of the 4%MS/PLLA scaffold. After 28 days, the mass of the PLLA scaffold decreased by 5.74 ± 1.38%, while the mass of the 4%MS/PLLA scaffold decreased by 5.50 ± 1.15%, with no significant difference between these two scaffolds (Supplementary Figure S7). The SEM images showed that after 7 days of incubation in SBF, all samples maintained their normal fiber structures, with most fibers displaying a smooth surface (Supplementary Figure S8). However, after 28 days of incubation, all the fibers became rough with a looser structure, especially in the 4%MS/PLLA scaffolds. Correspondingly, both the tensile strength and Young's modulus of the PLLA and 4%MS/PLLA groups decreased during the degradation process, although 4%MS/PLLA consistently exhibited higher values at all time points (Supplementary Figure S9). Furthermore, to assess ion release in the MS/PLLA composite scaffolds, concentrations of both Mg and Si ions were measured using the inductively coupled plasma mass spectrometry (ICP-MS) method. The results demonstrated that over time, concentrations of both Mg and Si gradually released from the composite scaffolds, reaching a plateau after 14 days (Figure 2F and G). All these findings suggest that MS/PLLA scaffolds degrade slowly, allowing for the continuous and gradual release of Mg and Si ions.

### MS/PLLA composite scaffold promotes the proliferation and differentiation of MuSCs

**Figure 3.**
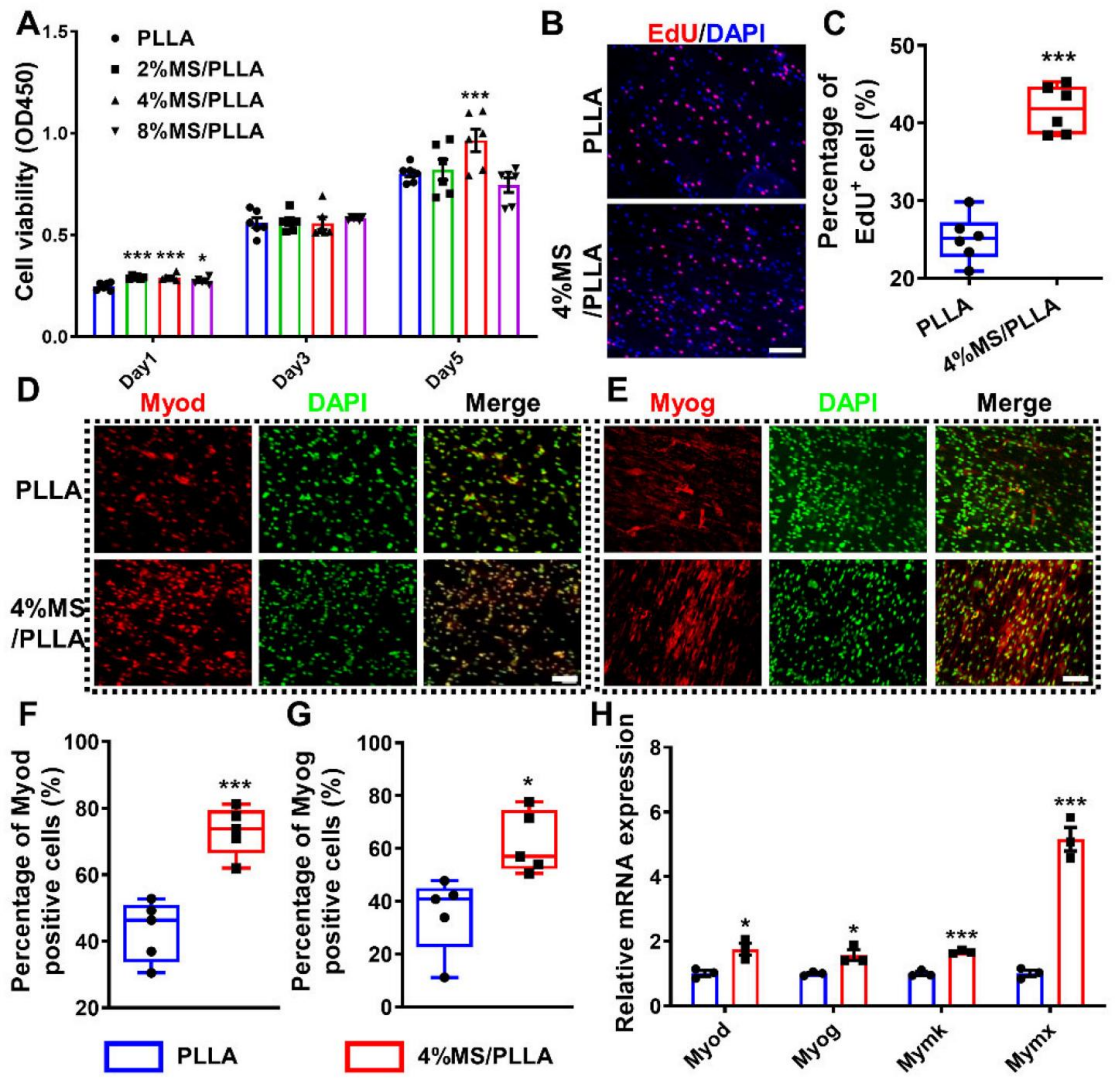
Effects of MS/PLLA composite scaffold on the proliferation and differentiation of MuSCs. (**A**) The cell viability of MuSCs on MS/PLLA composite scaffolds for 1, 3 and 5 days. (**B**) EdU staining of MuSCs after seeding for 48 h. Scale bar, 20 μm. (**C**). The percentage of EdU^+^ MuSCs in each group. (**D**). The Myod immunofluorescence staining results of MuSCs in each group. Scale bar, 50 μm. (**E**) The Myog immunofluorescence staining results of MuSCs in each group. Scale bar, 20 μm. (**F**) The percentage of Myod-positive MuSCs in each group. (**G**) The percentage of Myog-positive MuSCs in each group. (**H**) The relative m RNA expression of Myod, Myog, Mymk and Mymx in each group. *n* = 3. *P < 0.05, **P < 0.01, ***P < 0.001.

To investigate the effect of MS/PLLA composite scaffolds on MuSCs, MuSCs were cultured on the MS/PLLA composite scaffolds in growth medium and differentiated medium, respectively. CCK-8 assay results demonstrated that the cell viability of MuSCs in the 4%MS/PLLA group was significantly higher than in the other control groups (Figure 3A). Therefore, we chose 4%MS/PLLA as the representative composite in subsequent experiments. Additional CCK-8 assay revealed that compared with the control group, there was no significant difference in the viability of MuSCs in the PLLA group. However, cell viability in the 4%MS/PLLA group was significantly higher than that in the PLLA group (Supplementary Figure S10A). Live–dead assay results indicated no significant difference in the number of live and dead cells among all the groups, and MuSCs were able to effectively attach and grow along the orientation of both PLLA and 4%MS/PLLA scaffolds (Supplementary Figure S10B). These findings demonstrate that the MS/PLLA scaffold has good biocompatibility. Furthermore, the EdU staining results demonstrated that the percentage of EdU-positive MuSCs in the 4%MS/PLLA group was significantly higher than the PLLA group (Figure 3B and C). Besides, Myod immunofluorescence staining revealed that the percentage of Myod-positive MuSCs in the 4%MS/PLLA group was 1.69 times higher than that in the PLLA group (Figure 3D and F). Similarly, the percentage of Myog-positive MuSCs in the 4%MS/PLLA group was 1.77 times higher than that in the PLLA group (Figure 3E and G). The expression of the myogenic gene Myod and Myog in the 4%MS/PLLA group was both higher than the PLLA group (Figure 3H). Moreover, the expression of fusion genes Myomaker (Mymk) and Mymx in the 4%MS/PLLA group was also both higher than the PLLA group (Figure 3H). Overall, the Si ions and Mg ions released by 4%MS/PLLA significantly promote the proliferation, differentiation and fusion of MuSCs *in vitro*.

### MS/PLLA composite scaffold promotes muscle function recovery after VML

**Figure 4.**
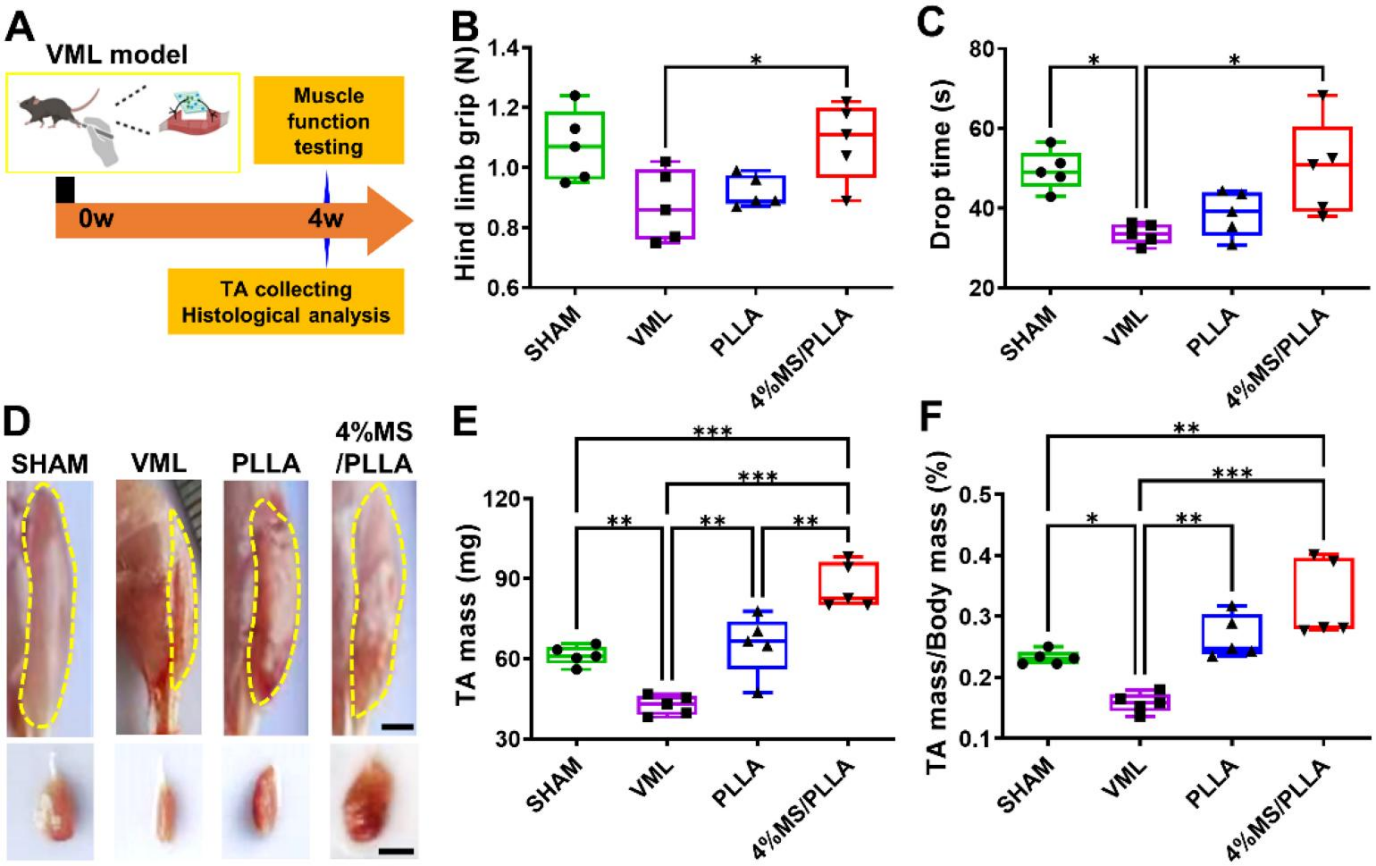
Effect of MS/PLLA composite scaffold on function and mass recovery of TA in VML model. (**A**) Schematic diagram of animal experiment flow. (**B**) The grip strength of the hind limb in different groups. *n* = 5. (**C**) The drop time of hind limb in different groups. *n* = 5. (**D**) The morphology of TA muscle in different groups at day 28. The scale bar for the first row of images is 2 mm, and the scale bar for the second row of images is 1 cm. (**E**) The mass of TA in different groups. (**F**) The ratio of TA mass/body mass in different groups. *n* = 5. *P < 0.05; **P < 0.01; ***P < 0.001.

To assess the impact of the prepared 4%MS/PLLA composite scaffolds on skeletal muscle injury, we utilized the VML mouse model in the TA and implanted the membrane at the injury site to investigate its efficacy in promoting regeneration and restoring muscle function (Figure 4A). The results demonstrated that the grip strength of the hind limb in the VML group was reduced, whereas a significant increase was observed in the 4%MS/PLLA group (Figure 4B). Similarly, the drop time in the VML group significantly decreased compared to the Shame group, while it increased in the 4%MS/PLLA group (Figure 4C). After 4 weeks of treatment, the TA muscle exhibited effective regeneration in the 4%MS/PLLA group compared to the VML and PLLA groups (Figure 4D). Additionally, the muscle mass of TA in the VML group decreased but was restored after both PLLA and 4%MS/PLLA treatment (Figure 4E and F). The muscle mass of TA in 4%MS/PLLA is slightly higher than that in the PLLA group. To investigate the degradation of the implanted scaffolds *in vivo,* both PLLA and 4%MS/PLLA scaffolds were taken out and photographed post-transplantation. All samples maintained a relatively intact scaffold morphology. Over time, an increasing amount of newly formed tissue, which was difficult to detach, was observed adhering to the material. This further demonstrates the stability of the materials and their potential for muscle tissue regeneration. (Supplementary Figure S11). Moreover, elevated levels of Mg and Si ions were observed in the local tissue adjacent to the 4%MS/PLLA scaffolds after administration for at least two weeks, suggesting the sustained release of Mg and Si ions from the composite scaffolds *in vivo* (Supplementary Figure S12). All these findings suggest that the Mg–Si ions combination effectively promotes TA muscle regeneration and restores muscle function.

### MS/PLLA composite scaffold promotes muscle fiber regeneration

**Figure 5.**
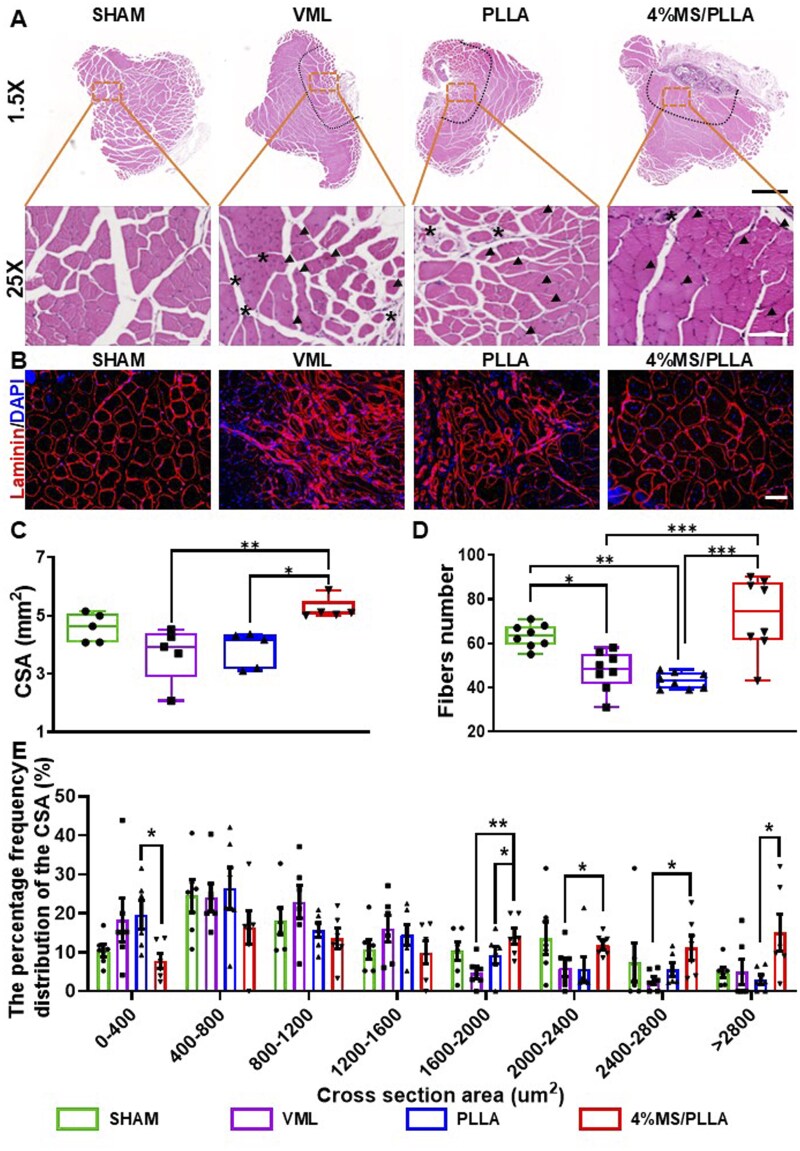
MS/PLLA composite scaffold accelerates the regeneration of muscle fiber. (**A**) H&E staining of TA muscle in each group. The scale bar for the first row of images is 1 mm, and the scale bar for the second row of images is 100 μm. The dotted line represents the interface between new tissue and normal muscle. The asterisk represents the area of inflammatory cell infiltration. The arrows indicate the myofibers with central nuclei. (**B**) Representative image of laminin immunofluorescence in each group. Scale bar: 50 μm. (**C**) Quantification result of the cross-section area of muscle fiber in each group. (**D**) Total number of muscle fibers in each group. (**E**) The distribution of CSA of TA muscle in each group. *, *P* < 0.05; **, *P* < 0.01; ***, *P* < 0.001.

In our study, H&E assay results demonstrated that revealed a reduced infiltration of inflammatory cells in the 4%MS/PLLA group compared to the VML and PLLA groups. the cross-section area (CSA) of TA muscle in the VML and PLLA groups was slightly lower than the control group, while the 4%MS/PLLA group was significantly higher than the VML and PLLA groups (Figure 5A and C). Additionally, immunofluorescence results for laminin demonstrated that showed a decrease in the total number of muscle fibers in the VML and PLLA groups, while a significant increase was observed in the 4%MS/PLLA group (Figure 5B and D). Furthermore, the percentage of muscle fiber with small CSA (0–400 μm^2^) in the VML and PLLA groups was more than in the Sham group, while that was decreased in the 4%MS/PLLA group. Conversely, the percentage of fiber with larger CSA (1600–2800 μm^2^) in the VML and PLLA groups was less than the Sham group, while that was significantly increased in the 4%MS/PLLA group compared with the other VML and PLLA groups (Figure 5E). Above all, the Mg–Si ions combination facilitated the regeneration of muscle fiber *in vivo*.

**Figure 6.**
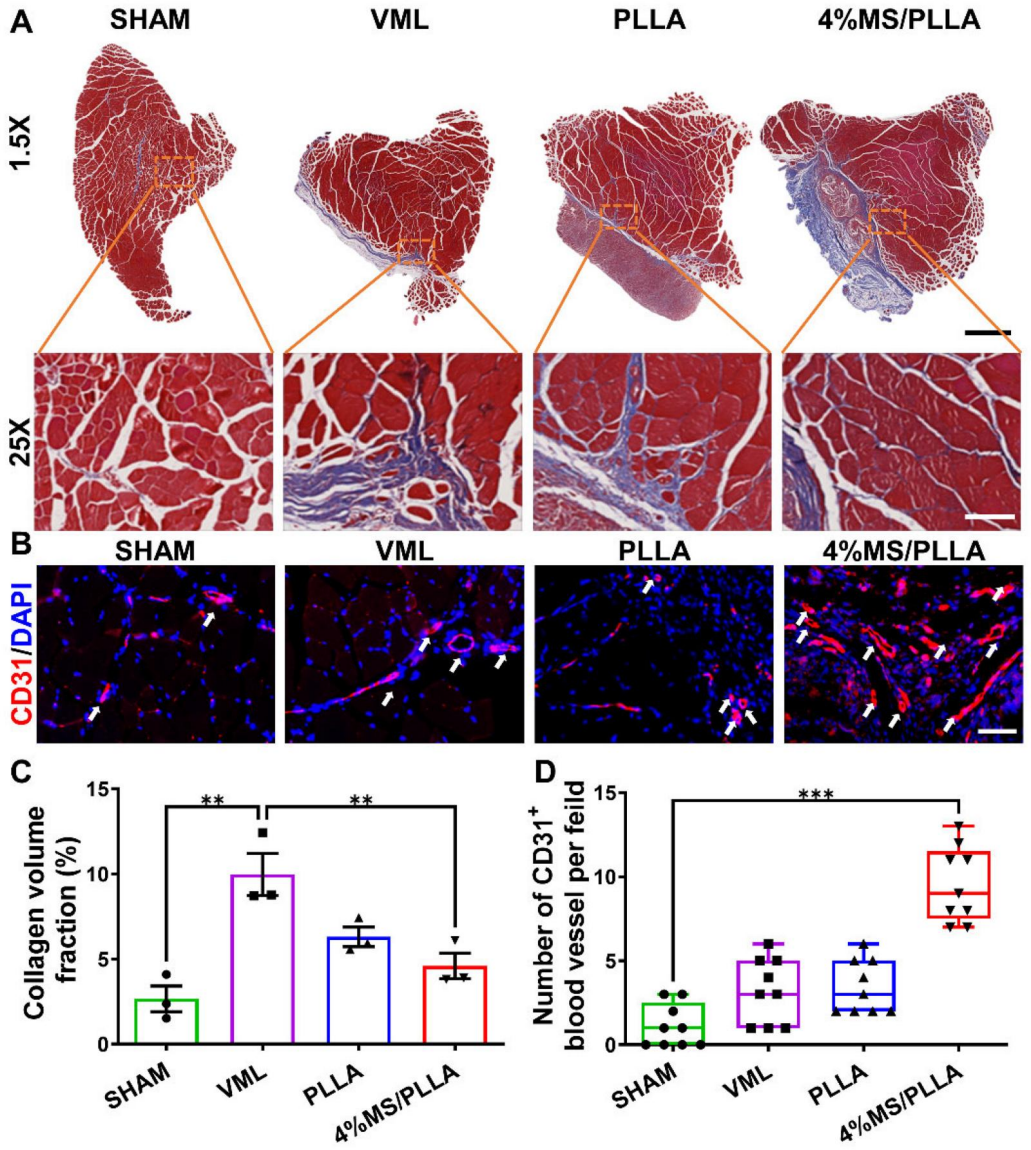
MS/PLLA composite scaffold alleviates fibrosis but promotes neovascularization. (**A**) Masson staining of TA muscle in each group. The scale bar for the first row of images is 1 mm, and the scale bar for the second row of images is 100 μm. (**B**) Representative image of CD31 immunofluorescence in each group. Scale bar: 50 μm. (**C**) Quantification result of collagen volume fraction in each group. (**D**) Number of CD31 positive blood vessels per field in each group. White arrows represent CD31-positive blood vessels. *, *P* < 0.05; **, *P* < 0.01; ***, *P* < 0.001.

Masson staining results indicated that a reduced fraction of collagen volume in TA muscle was reduced in the 4%MS/PLLA group compared with the other control groups, suggesting that 4%MS/PLLA composite scaffold treatment effectively alleviated the fibrosis of TA muscle (Figure 6A and C). Additionally, the CD31 immunofluorescence results demonstrated a significant increase in the number of CD31-positive blood vessels in the 4%MS/PLLA group compared with the other control groups (Figure 6B and D). Above all, the 4%MS/PLLA composite scaffold appears to promote the regeneration of skeletal muscle fibers potentially by attenuating fibrosis and fostering neovascularization. Taken together, the Mg–Si ions combination effectively promotes the regeneration of muscle fiber and neovascularization, while alleviating fibrosis, thus facilitating the repair of skeletal muscle injury.

### MS/PLLA composite scaffold promotes the activation and differentiation of MuSCs

**Figure 7.**
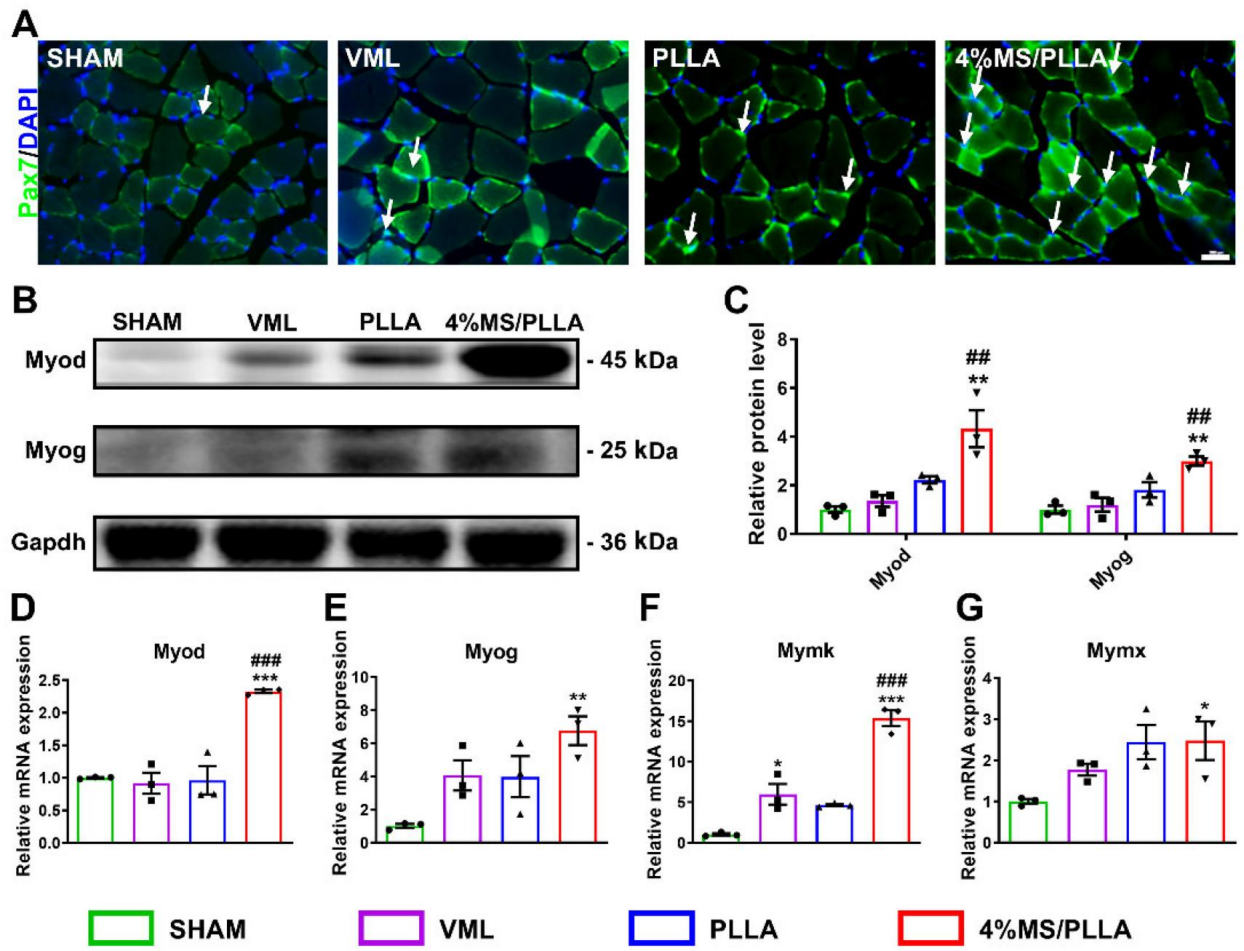
MS/PLLA composite scaffold promotes the activation, differentiation and fusion of MuSCs *in vivo*. (**A**) Immunofluorescence staining of Pax7 in TA. Scale bar: 100 μm. The white arrows represent the Pax7-positive activated MuSCs. (**B**) Western blot results of Myod and Myogenin in each group. (**C**) Statistics results of the protein level of Myod and Myog in each group. The relative mRNA expression of Myod (**D**), Myog (**E**), Mymk (**F**), Mymx (**G**) in each group. *n* = 3. * represents comparison with the Sham group, # represents comparison with the VML group. *, *P* < 0.05; ** and ##, *P* < 0.01; *** and ###, *P* < 0.001.

Prior research has established the crucial role of Pax7-positive skeletal MuSCs in the process of regenerating injured skeletal muscle [38]. Immunofluorescence results of Pax7 demonstrated that the number of Pax7-positive MuSCs in the 4%MS/PLLA group was significantly higher than in the other control groups (Figure 7A and Supplementary Figure S13). Besides, the protein levels of myogenic factor Myod and Myog in the 4%MS/PLLA group were both higher compared with the other control groups (Figure 7B and C). Similarly, the mRNA levels of Myod and Myog in the 4%MS/PLLA group were both higher than the control group (Figure 7D and E). Moreover, the mRNA level of fusion genes Mymk and Mymx was both higher in the 4%MS/PLLA group compared with the other control groups (Figure 7F and G). Taken together, the Mg–Si ions combination can promote the activation and differentiation of MuSCs *in vivo* to accelerate the regeneration of skeletal muscle.

### MS/PLLA composite scaffold improves the activation and proliferation of MuSCs via up-regulating the Notch signal pathway

**Figure 8.**
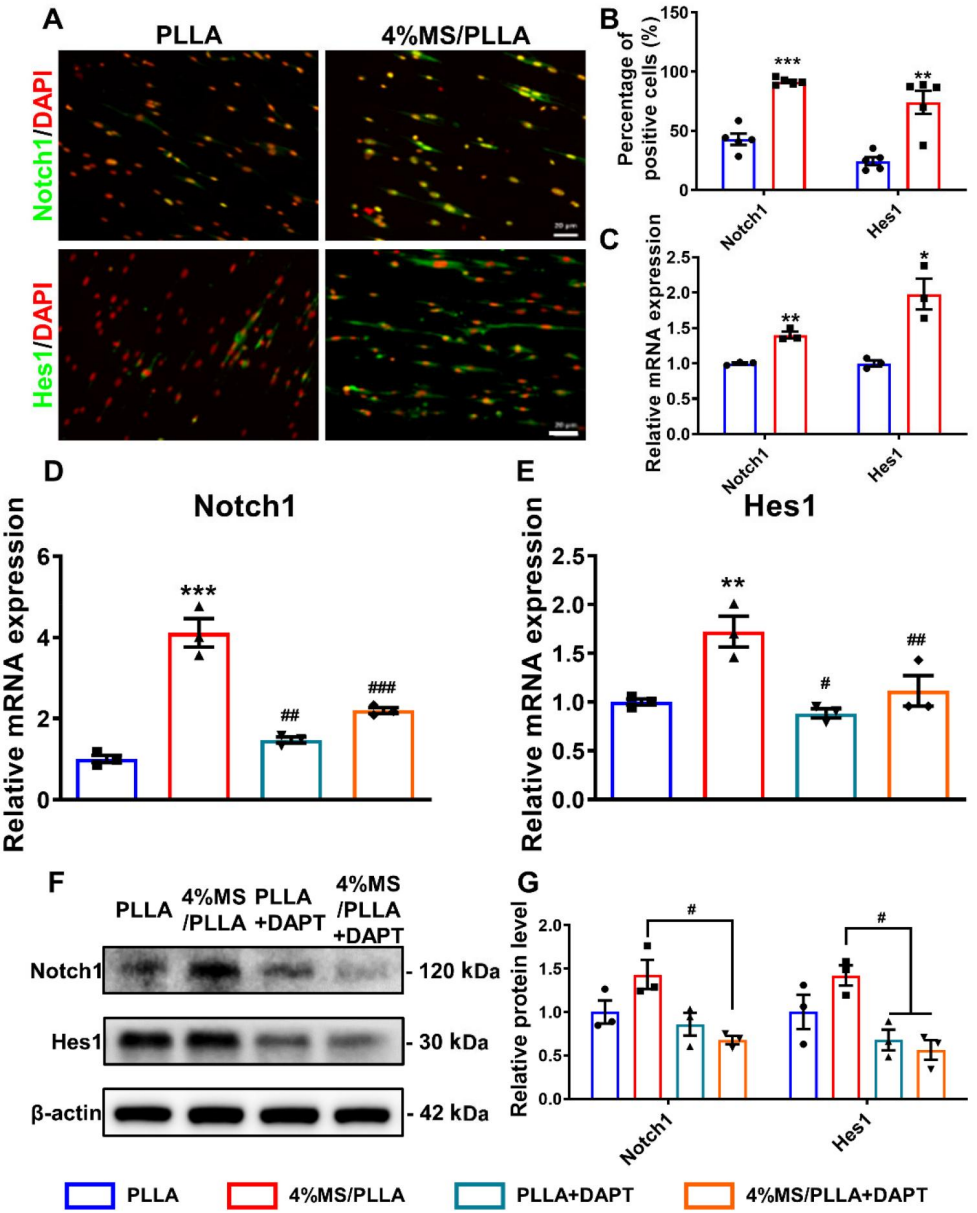
MS/PLLA composite scaffold promotes the activation and proliferation of MuSCs by activating the Notch1–Hes1 signal axis. (**A**) Immunofluorescence staining of Notch1 and Hes1 in MuSCs cocultured with PLLA and 4%MS/PLLA, respectively. Scale bar, 20 μm. (**B**) The percentage of Notch1 and Hes1 positive MuSCs in each group. (**C**) The relative mRNA expression of Notch1 and Hes1 in each group. (**D**) The relative mRNA expression of Notch1 in each group. (**E**) The relative mRNA expression of Hes1 in each group. DAPT, γ-secretase inhibitor. (**F**) The relative protein expression of Notch1 and Hes1 in each group. (**G**) Statistics results of the protein level of Notch1 and Hes1 in each group. * represents comparison with PLLA group, # represents comparison with the 4%MS/PLLA group. * and #, *P* < 0.05; ** and ##, *P* < 0.01; *** and ###, *P* < 0.001.

Furthermore, we examined the expression of the Notch signaling pathway in MuSCs treated with 4%MS/PLLA *in vitro*. Immunofluorescence results demonstrated that 4%MS/PLLA loading also upregulated the expression of Notch1 and Hes1 (Figure 8A and B). Similarly, the mRNA levels of Notch1 and Hes1 in 4%MS/PLLA were both significantly higher than in the PLLA group (Figure 8C). To confirm whether the regulatory effect of Mg–Si ions combination on MuSCs is mediated by Notch signaling, we used the Notch1 blocker γ-secretase inhibitor (DAPT) to block Notch1 for further verification. The results demonstrated that Notch1 blocking inhibited the expression of downstream Hes1. Moreover, compared with the 4%MS/PLLA group_,_ blocking Notch1 significantly inhibited the upregulation of Notch1 and downstream Hes1 in MuSCs treated with 4%MS/PLLA (Figure 8D and E). Consistent with the qPCR experiment results, the relative protein levels of Notch1 and Hes1 in the 4%MS/PLLA group were higher than those in the PLLA group. However, blocking Notch1 significantly inhibited the increased protein expression of both Notch1 and Hes1 in MuSCs treated with 4%MS/PLLA (Figure 8F and G). Taken together, we suspect that the Mg and Si ions released from 4%MS/PLLA promote the activation and proliferation of MuSCs by specifically activating the Notch1-Hes1signal pathway.

## Discussion

The activation of MuSCs is crucial for muscle regeneration. The extracellular matrix (ECM) at the injury site releases growth factors that interact with satellite cells, stimulating their activation. Compared to healthy muscle, both the number and activity of satellite cells significantly increase in damaged muscle. Activated satellite cells can traverse barriers of the basal lamina and connective tissue, migrating between muscle fibers and even across muscles [39]. Additionally, these cells undergo division to provide sufficient progeny to form new muscle fibers [40]. Previous research by Awad *et al.* found that Si ions can enhance the activity and proliferation of myogenic C2C12 cells [25]. However, the regulatory effects of Si ions on MuSCs have not been reported. In this study, we demonstrate for the first time that Si ions increase the viability and proliferation of MuSCs. Mg ions, on the other hand, do not significantly affect MuSC viability and proliferation, consistent with findings by Liu *et al.* in myogenic cells [23]. Interestingly, the proportion of EdU-positive MuSCs was 1.23 times higher with the combined addition of Mg and Si ions compared to Si ions alone and 1.35 times higher compared to Mg ions alone. Similarly, MuSCs viability was 1.19 times higher with the combined ions than with Si ions alone and 1.40 times higher than with Mg alone. Our study is the first to show that the combined addition of Mg and Si ions synergistically enhances MuSCs proliferation and viability. Pax7 is a key regulator of MuSCs activation and self-renewal [41, 42]. We demonstrated that 4%MS/PLLA composite scaffolds, which possessed the ability to sustained release Mg and Si ions, significantly increase the number of Pax7-positive cells at the injury site following VML. In summary, our research provides the first evidence that the combination of Mg and Si ions further promotes MuSCs activation and proliferation.

During skeletal muscle regeneration, after several rounds of proliferation, most MuSCs differentiate into myogenic precursor cells or mature myogenic cells, providing the cellular source for regenerating damaged muscle fibers, with only a small fraction returning to a quiescent state. Previous studies have shown that Si ions can promote myogenic cell differentiation and myotube formation [25, 29]. Additionally, Mg ions have been found to significantly enhance myogenic cell differentiation into myotubes [23]. In our study, we discovered that Mg ions also promote the differentiation and myotube formation of skeletal MuSCs. More importantly, we found for the first time that the combined Mg and Si ions synergistically enhance the differentiation of MuSCs. During MuSCs differentiation, the expression of MyoD is crucial for their myogenic differentiation [43], while Myogenin expression is key for MuSCs to exit the cell cycle and form multinucleated muscle fibers [44]. In our study, we found that the addition of either Mg or Si ions alone upregulated MyoD and Myogenin expression in MuSCs, and their combined addition showed an even stronger synergistic effect, further increasing the proportion of Myosin-positive multinucleated myotubes. In the Mg–Si combination group, the proportion of Myosin-positive multinucleated myotubes was 1.68 times higher than in the Si group alone and 1.69 times higher than in the Mg group alone. Furthermore, in the VML model, the expression levels of MyoD and Myogenin in the 4%MS/PLLA composite scaffold group were significantly higher than in the PLLA group, further demonstrating the potential of the Mg and Si ions combination to promote myogenic differentiation and skeletal muscle regeneration *in vivo*.

Considering that cell fusion is a critical step in myogenic cells repairing damaged muscle fibers, recent studies have shown that the muscle cell-specific fusion proteins Mymk and Mymx play key roles in regulating the fusion process in myogenic cells. Specifically, Mymk is a crucial membrane protein regulating myogenic cell fusion during embryonic and adult skeletal muscle regeneration [45]. After skeletal muscle injury, basic helix-loop-helix (bHLH) transcription factors induce Mymk expression in skeletal MuSCs, promoting myogenic cell fusion and muscle regeneration [46]. Mymx is another muscle-specific membrane micropeptide that regulates myogenic cell fusion [47]. During skeletal muscle regeneration, Mymk and Mymx sequentially regulate the progressive fusion of myogenic cells, thereby promoting muscle regeneration [48]. In the study, we found that the combination of Mg and Si ions significantly upregulated the expression of Mymk and Mymx in MuSCs cultured *in vitro*. This combination also increased the expression of Mymk and Mymx during skeletal muscle regeneration, promoting the fusion of myofibers after muscle injury. This study is the first to demonstrate the positive regulatory effects of the Mg–Si ions combination on myogenic cell–myogenic cell fusion as well as myogenic cell–myofiber fusion. In conclusion, our research provides the first evidence that a specific combination of Mg and Si ions positively regulates MuSC activation, proliferation, differentiation and fusion, as well as skeletal muscle regeneration. Understanding the underlying mechanisms behind these observations is crucial for advancing therapeutic strategies.

Notch signaling plays a crucial regulatory role in maintaining MuSC quiescence, activation, proliferation and differentiation processes [49]. Following skeletal muscle injury, the Notch ligand Delta is rapidly increased in activated satellite cells and muscle fibers [50]. Specific overexpression of the intracellular fragment of Notch1 in MuSCs enhances the activation of Pax7-positive skeletal MuSCs [51]. Moreover, overexpression of the intracellular fragment of Notch1 effectively promotes the proliferation of porcine skeletal MuSCs by up-regulating Hes1 and Cyclins [52]. In our study, 4%MS/PLLA treatment increased the activation of Pax7-positive MuSCs and upregulated the expression of Notch1 and Hes1 in the TA muscle. *In vitro*, 4%MS/PLLA loading increased the expression of Notch1, Hes1 and cell cycle proteins, thereby promoting MuSCs proliferation. Additionally, blocking Notch signaling with DAPT inhibited the upregulation of Notch1 and Hes1, as well as downstream cell cycle proteins Ccnb1 and Ccnd1 in all the groups. These results collectively suggest that the Mg–Si ions combination synergistically activates the Notch1–Hes1–cyclins axis, thereby promoting the functional role of skeletal MuSCs in muscle regeneration.

**Figure 9.**
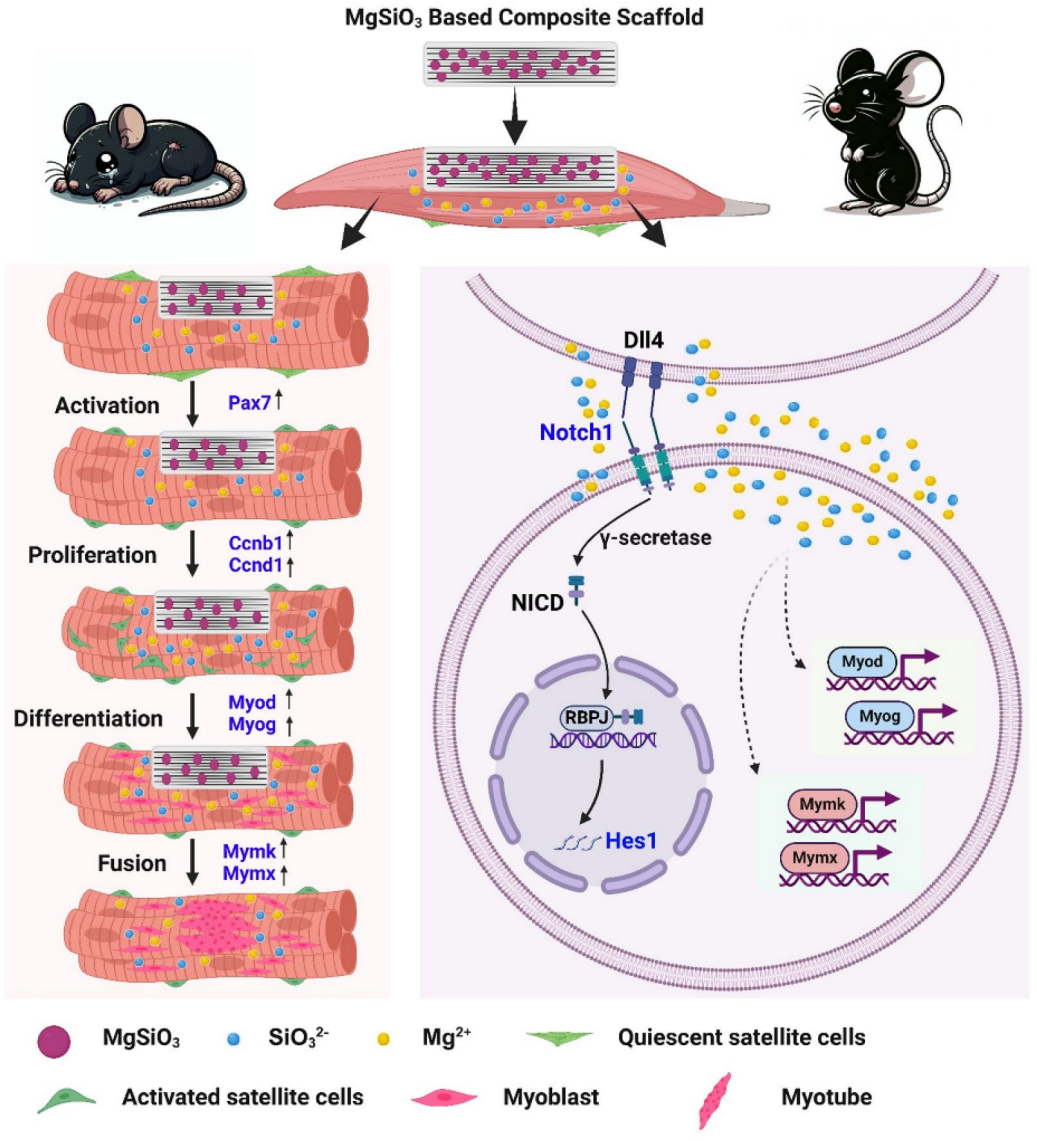
Synergistic promotion of skeletal muscle functional regeneration by the combined Mg–Si ions strategy. The combination of Mg and Si ions enhances the activation and proliferation of MuSCs via the Notch1–Hes1 signaling axis. Additionally, this combination promotes MuSCs differentiation by up-regulating Myod and Myog expression. Importantly, it also increases myoblast fusion by boosting the expression of Mymk and Mymx. Overall, the Mg–Si ions combination effectively accelerates functional regeneration of skeletal muscle by synergistically regulating multiple steps in the regeneration process, including MuSC activation, proliferation, differentiation and fusion.

Furthermore, we analysed the advantages and limitations of the Mg–Si ions therapy strategy in regulating MuSCs and promoting muscle regeneration. Specifically, its advantages include several aspects: Mg–Si ions combination can synergistically enhance the cellular activity and proliferation of skeletal MuSCs; it can synergistically promote the differentiation and fusion of MuSCs to form multinucleated myotubes; additionally, Mg and Si ions can promote skeletal muscle regeneration by regulating collagen deposition, and new blood vessel formation (Figure 9). Unfortunately, this study was unable to effectively monitor the release and distribution of Mg and Si ions *in vivo*. In addition, the Notch1 signaling pathway was not blocked *in vivo* to explore the regulatory mechanism of Notch1 signaling in the synergistic promotion of skeletal muscle regeneration by Mg and Si ions. Besides, we only detected the distribution and quantity differences of macrophages at the early stage of skeletal muscle injury sites and did not delve into the regulatory mechanisms of the Mg–Si ions combination on inflammatory factors and immune responses. These limitations need further experimental design to verify and explore. In summary, this study will provide a new ion combination therapy strategy for refractory muscle diseases.

## Conclusion

In this study, we first found that the Mg–Si ions combination can synergistically promote the activation and proliferation of MuSCs *via the* Notch1–Hes1 pathway. Besides, the Mg–Si ions combination facilitates the differentiation of MuSCs by up-regulating the expression of Myod and Myog. More importantly, the Mg–Si ions combination increases the fusion of myoblasts by improving the expression of Mymk and Mymx. Overall, the Mg–Si ions combination effectively accelerates the functional regeneration of skeletal muscle by synchronously regulating multiple steps of MuSCs during the process of skeletal muscle regeneration, including MuSCs activation, proliferation, differentiation and fusion.

## Supplementary Material

rbaf008_Supplementary_Data
