# Supplementary material for: Combined magnesium and silicon ions synergistically promote functional regeneration of skeletal muscle by regulating satellite cell fate
Source: Regen Biomater. 2025 Feb 19;12:rbaf008. doi: 10.1093/rb/rbaf008 (PMC11932723; doi:10.1093/rb/rbaf008)
Supplement: rbaf008_Supplementary_Data [file rbaf008_supplementary_data.docx]

**Combined** **Magnesium and Silicon Ions Synergistically Promote Functional Regeneration of Skeletal Muscle by Regulating Satellite Cell Fate**

*Hangbin Xia,* *Chen Yang, Huili Li, Lingwei Huang, Zhen Zeng, Runrun Chi, Ziwei Yang, Yuzen Wang, Jiang Chang, Yiren Jiao, Wenzhong Li*

**Supplementary Information**

**Contents of Supplementary Information**

**Figure S1:** Identification of skeletal muscle satellite cells.

**Figure S2:** Immunofluorescence staining of Myod in MuSCs after 3 days of differentiation.

**Figure S3:** Screening of magnesium ions concentrations.

**Figure S4**: Screening of silicon ions concentrations.

**Figure S5**: Orientation degree of MS/PLLA composite scaffold.

**Figure S6**: Mechanical properties of MS/PLLA composite scaffolds.

**Figure S7:** Degradation of PLLA and 4% MS/PLLA scaffolds in simulated body fluids.

**Figure S8:** The morphology of PLLA and 4%MS/PLLA scaffolds during degradation in simulated body fluids.

**Figure S9:** Mechanical properties of PLLA and 4%MS/PLLA scaffolds in simulated body fluids.

**Figure S10:** Biocompatibility of PLLA and 4%MS/PLLA scaffolds.

**Figure S11:** Photographs of the implanted scaffolds at different time points in vivo.

**Figure S12:** Increased concentrations of Mg and Si ions in local TA muscles.

**Figure S13:** Statistical results of the number of Pax7-positive MuSCs in the TA muscle of each group.

**Supplementary Table S1:** Primer sequences used for qRT-PCR.

**Supplementary Table S2:** Information of antibodies used in this study.


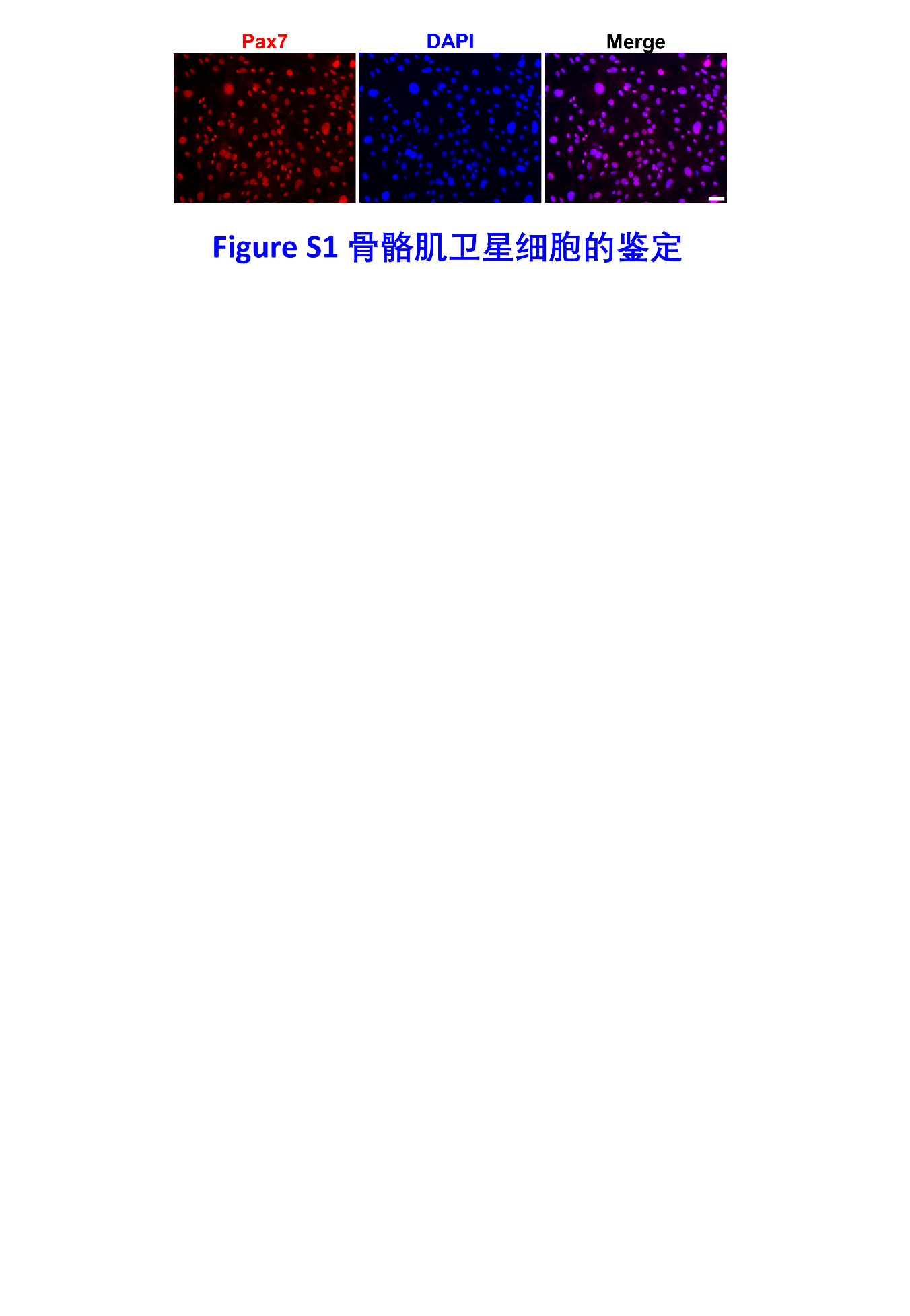


**Figure S1. Identification of skeletal muscle satellite cells.** Immunofluorescence staining of Pax7 in MuSCs, Scale bar: 20 μm.


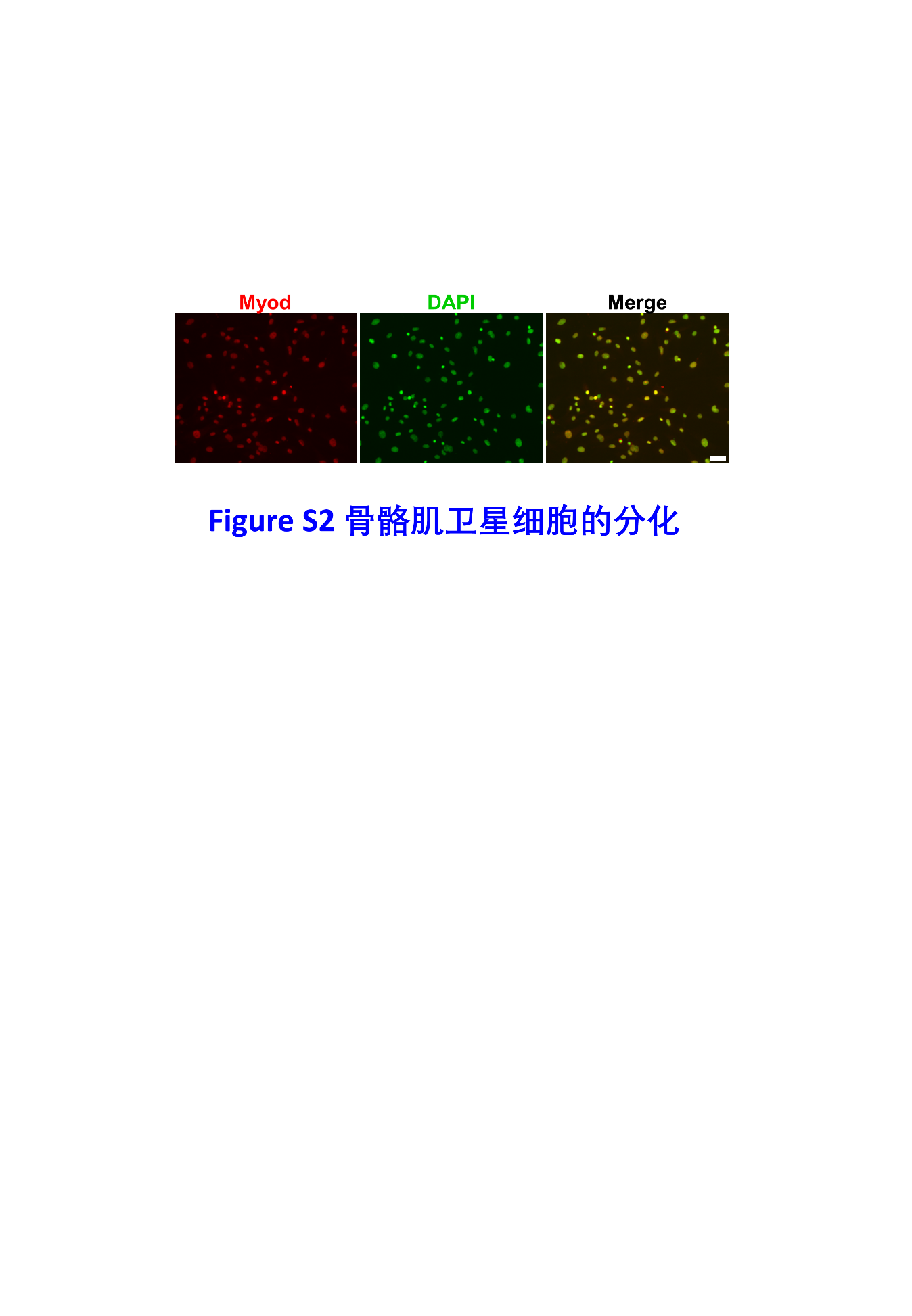


**Figure S2. Immunofluorescence staining of Myod in MuSCs after 3 days of differentiation.** Scale bar: 20 μm.


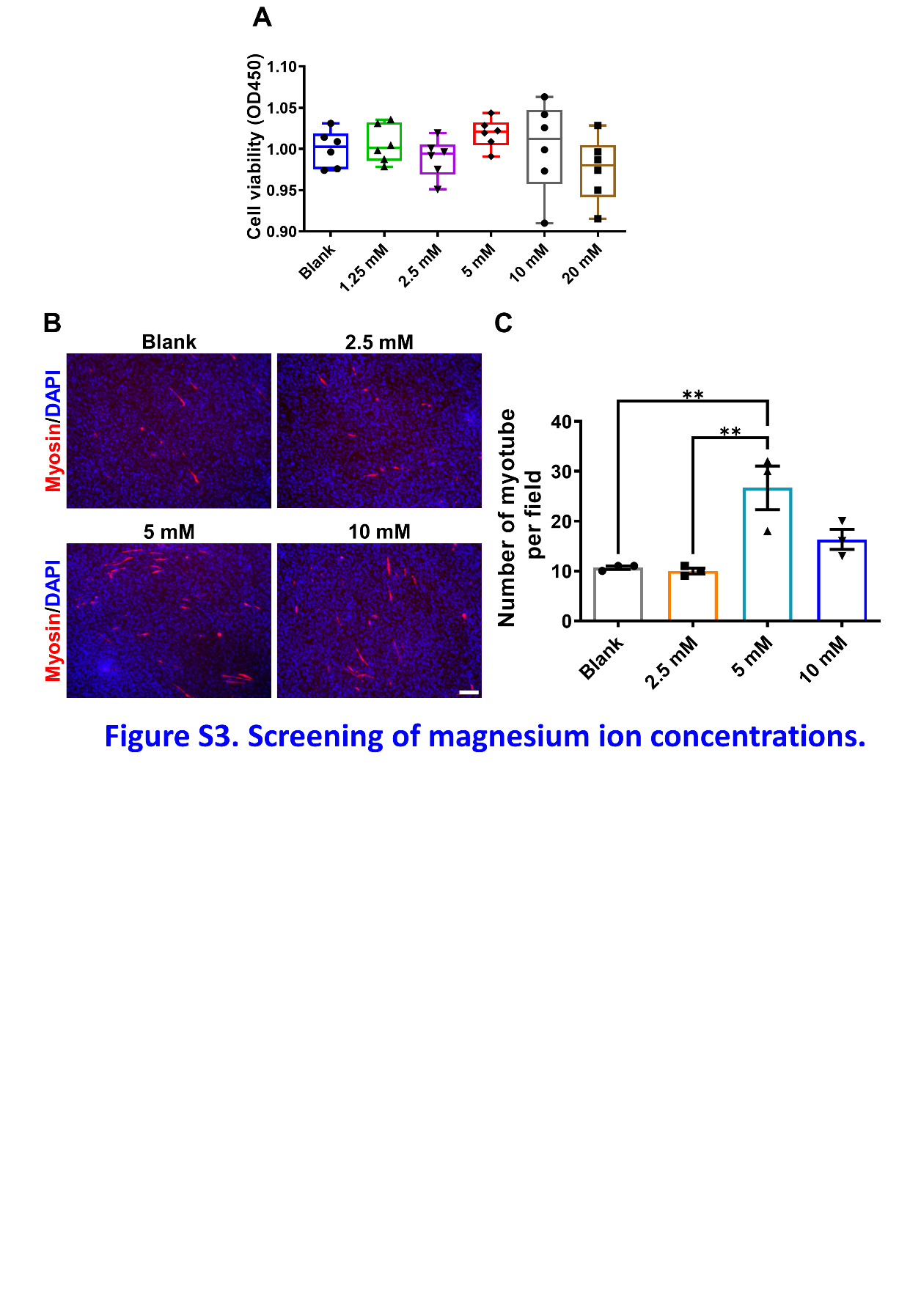


**Figure S3. Screening of** **magnesium ions concentrations.** A. CCK-8 assay showing cell viability of MuSCs treated with different concentrations of magnesium ions. B. Immunofluorescence staining of Myosin in MuSCs treated with various concentrations of magnesium ions. Scale bar: 100 μm. C. The number of Myosin-positive myotubes per field in each group (n=3). **, p<0.01.


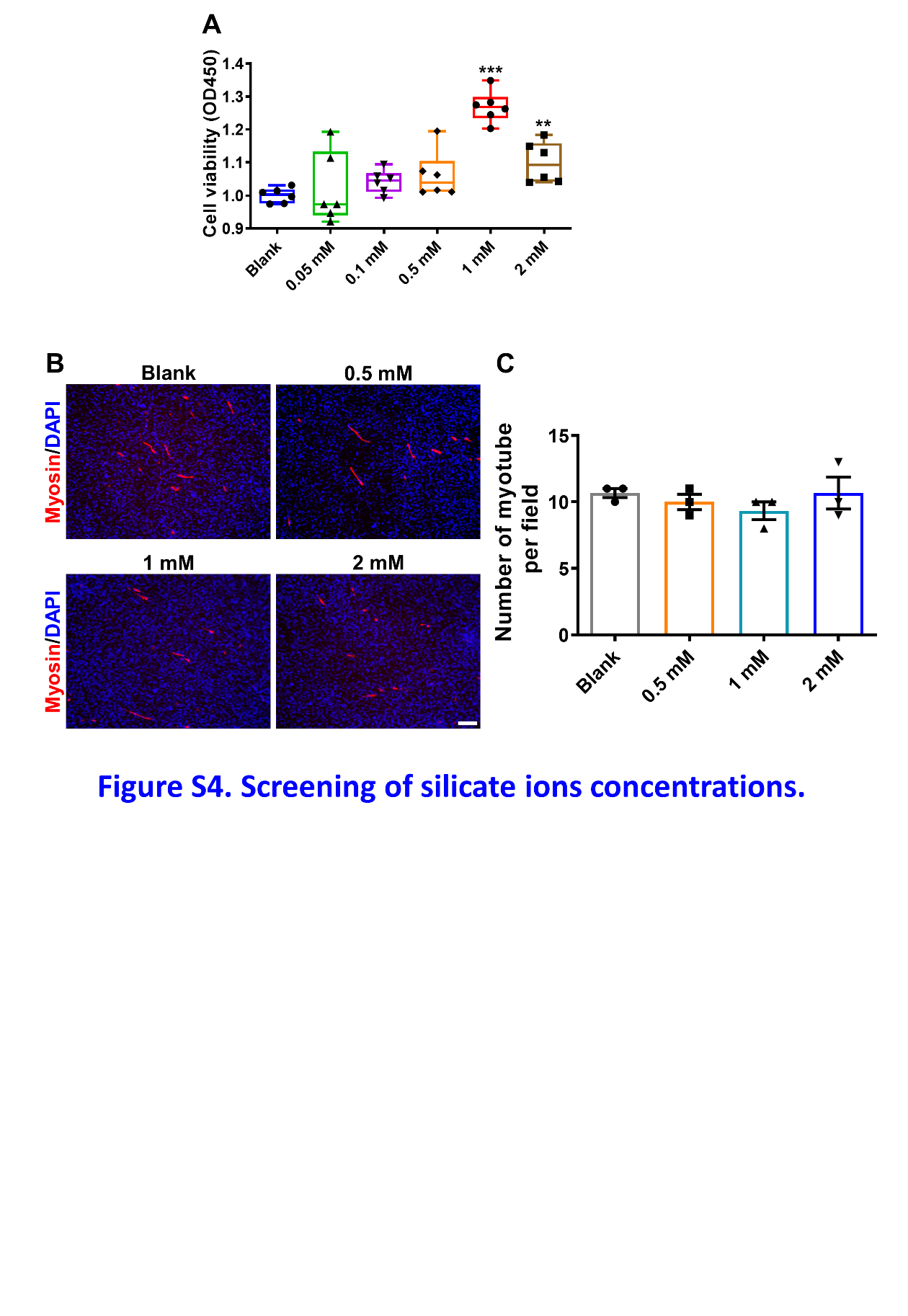


**Figure S4**. **Screening of** **silicon** **ions concentrations.** A. CCK-8 assay showing cell viability of MuSCs treated with different concentrations of silicon ions. B. Immunofluorescence staining of Myosin in MuSCs treated with various concentrations of silicon ions. Scale bar: 100 μm. C. The number of Myosin-positive myotubes per field in each group (n=3). **, p<0.01. ***, p<0.001.


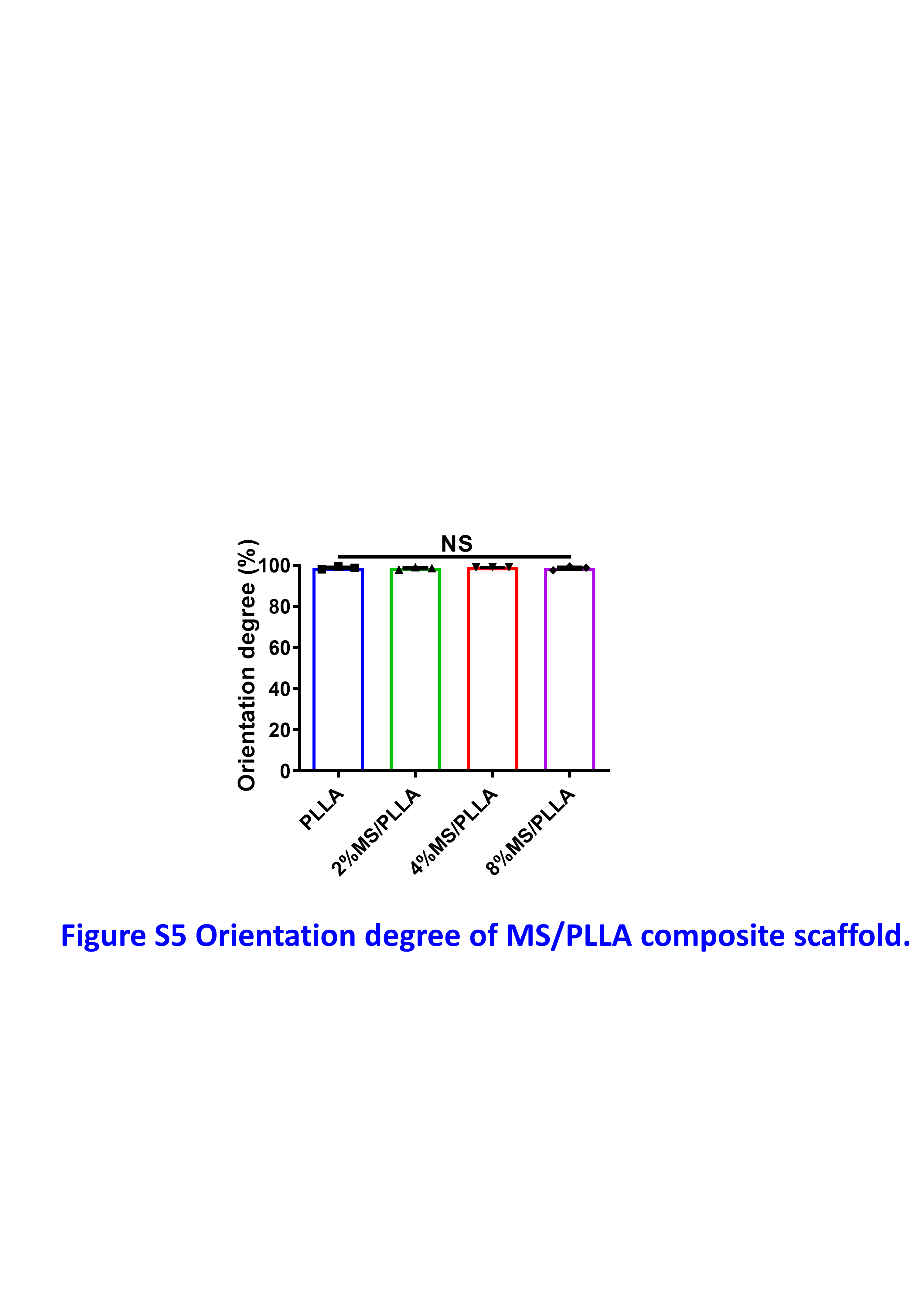


**Figure S5**. **Orientation degree of MS/PLLA composite scaffold.**


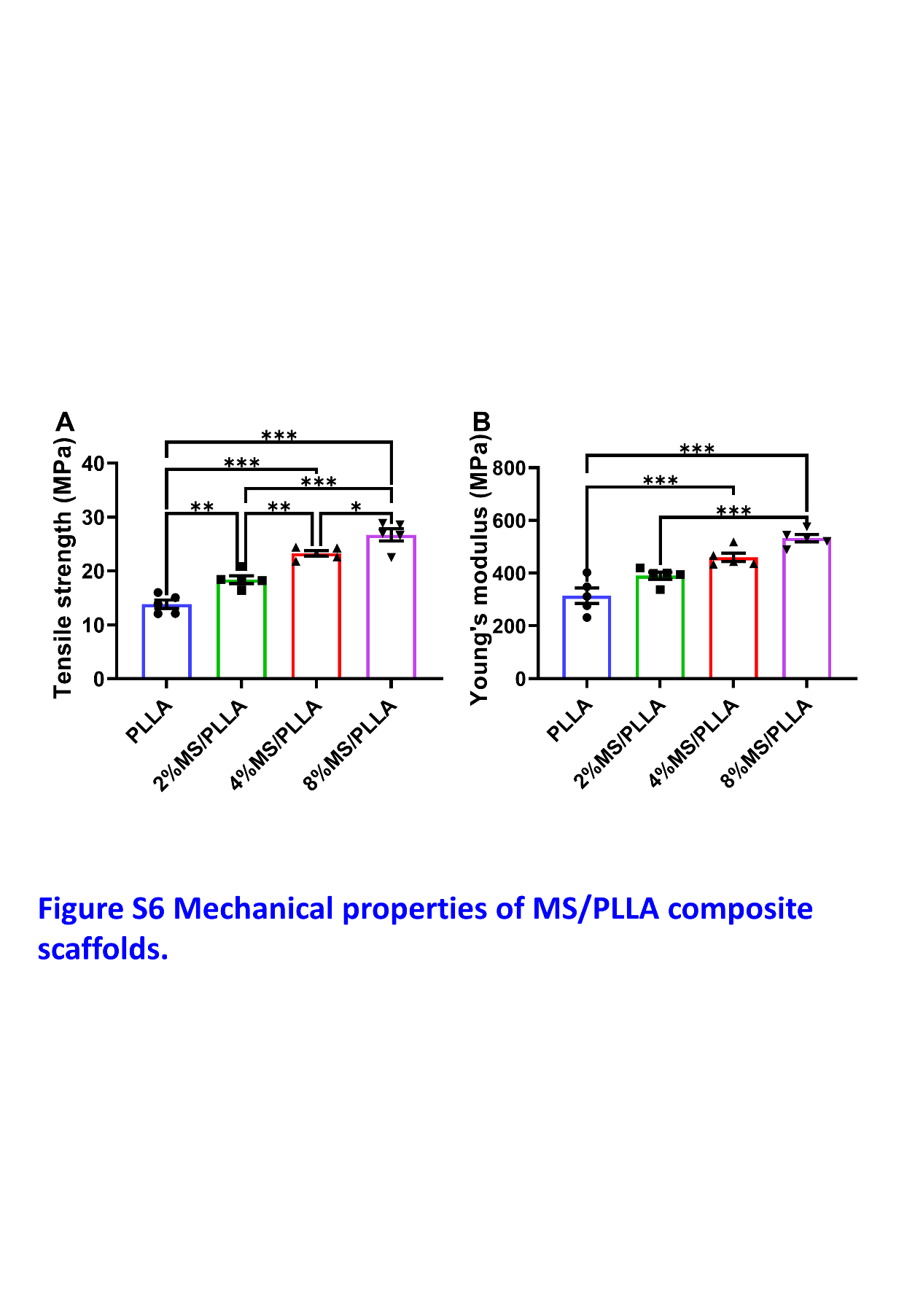


**Figure S6**. **Mechanical properties of MS/PLLA composite scaffolds.** A. Tensile strength of MS/PLLA scaffolds. B. Young's modulus of MS/PLLA scaffolds. n=5. *, p<0.05; **, p<0.01; ***, p<0.001.


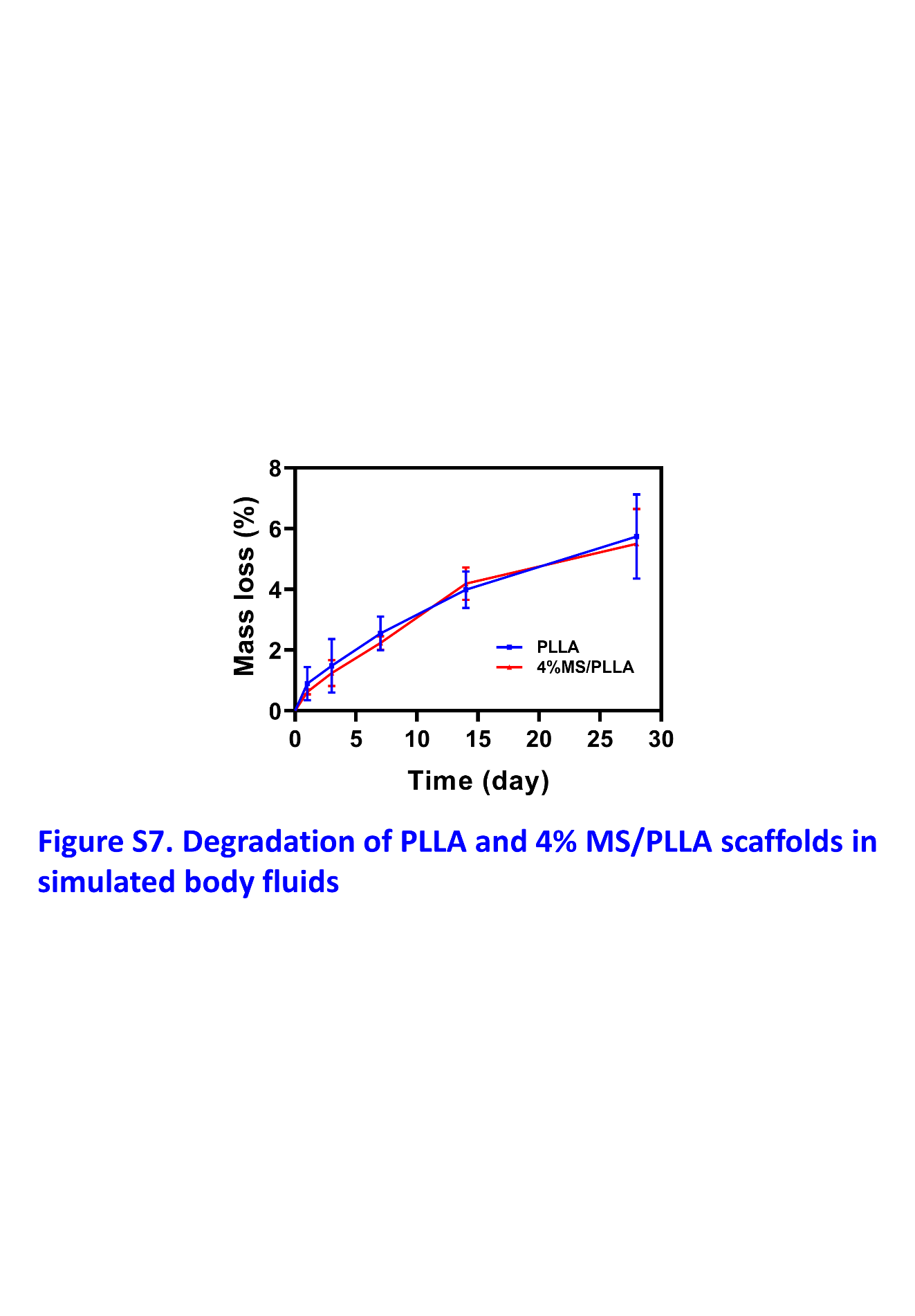


**Figure S7. Degradation of PLLA and 4% MS/PLLA scaffolds in simulated body fluids.**

**
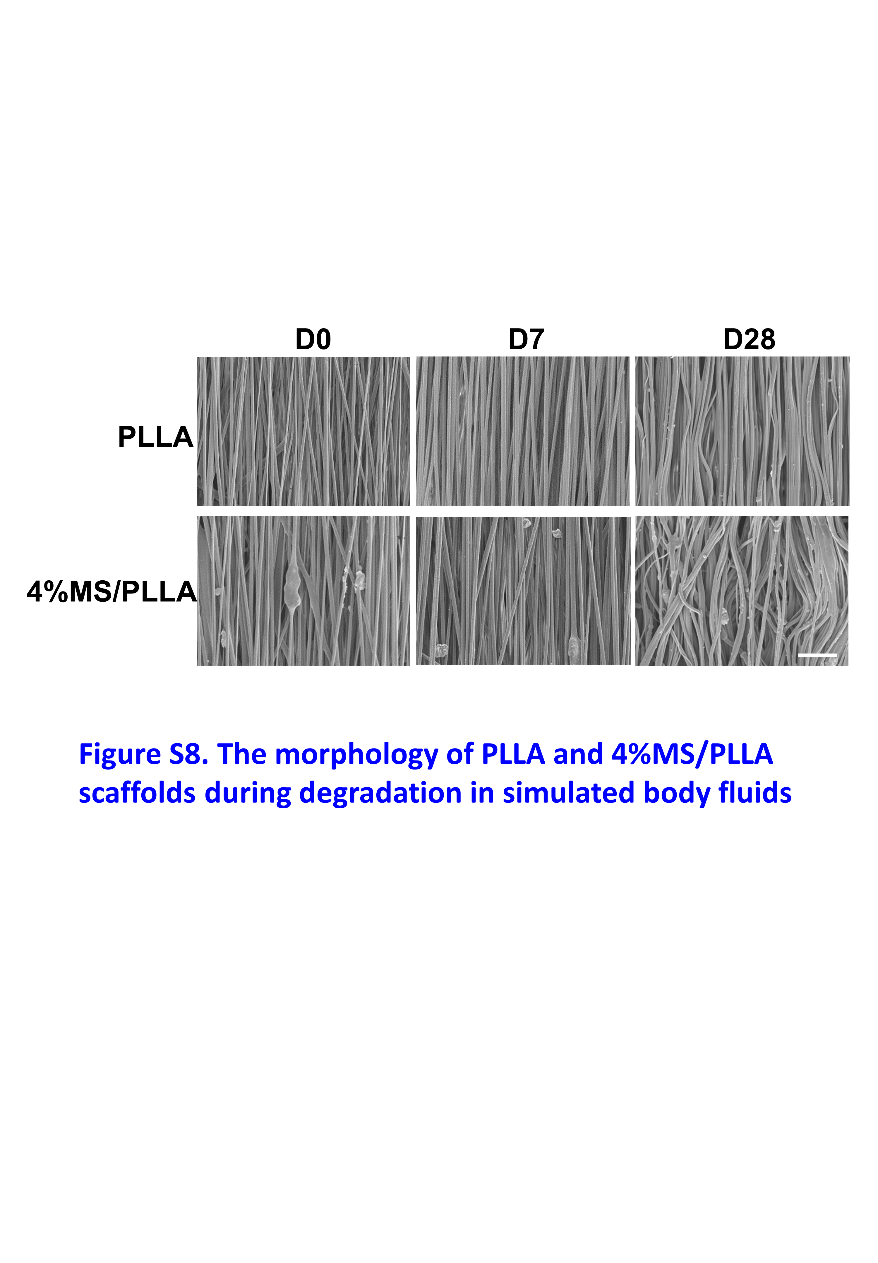
**

**Figure S8. The morphology of PLLA and 4%MS/PLLA scaffolds during degradation in simulated body fluids.** Scale bar: 10 μm.

**
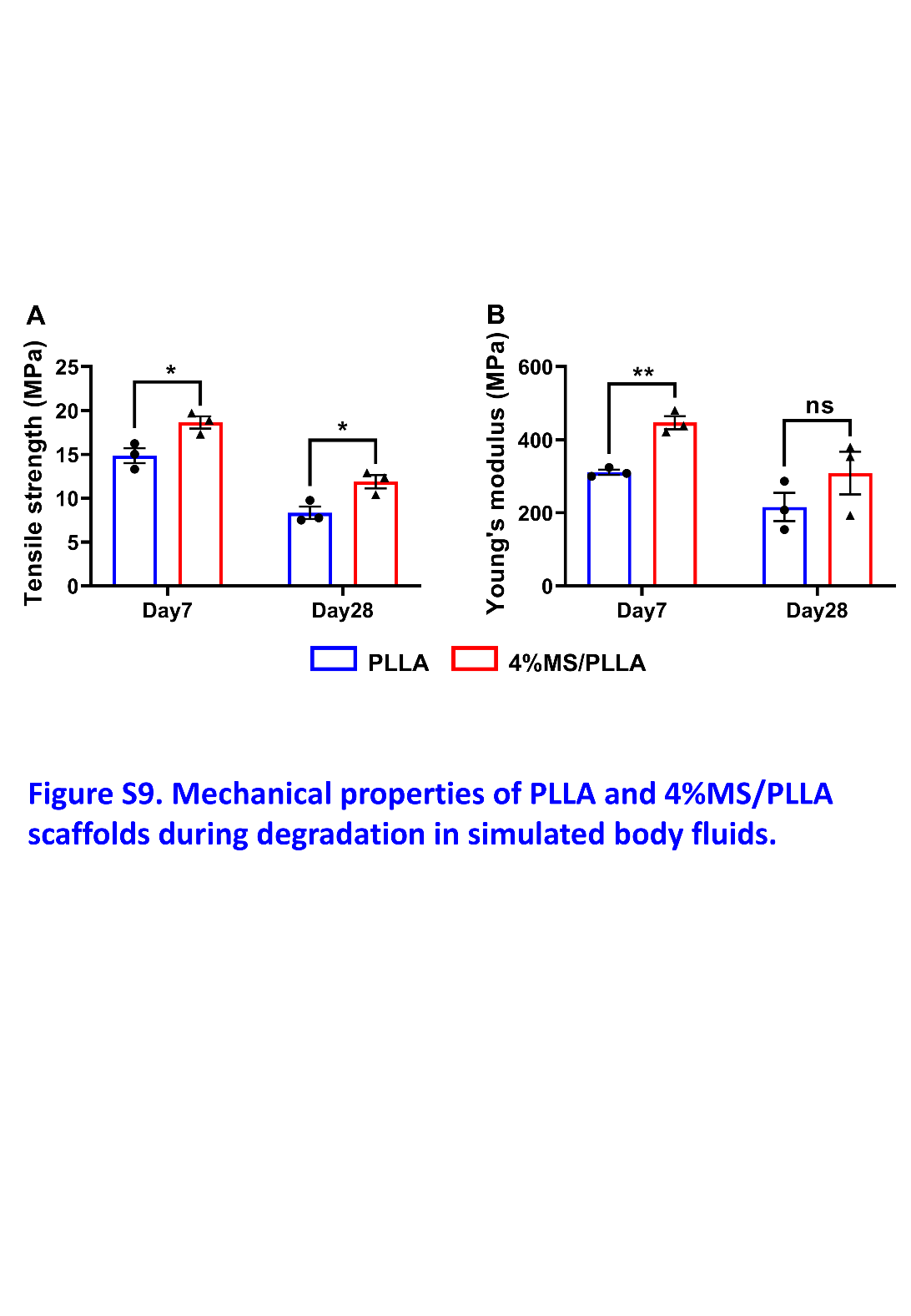
**

**Figure S9. Mechanical properties of PLLA and 4%MS/PLLA scaffolds in simulated body fluids.** A. Tensile strength of PLLA and 4%MS/PLLA scaffolds degraded in SBF for 7 days and 28 days, respectively. B. Young’s modulus of PLLA and 4%MS/PLLA scaffolds degraded in SBF for 7 days and 28 days, respectively. n=3. *, p<0.05; **, p<0.01.


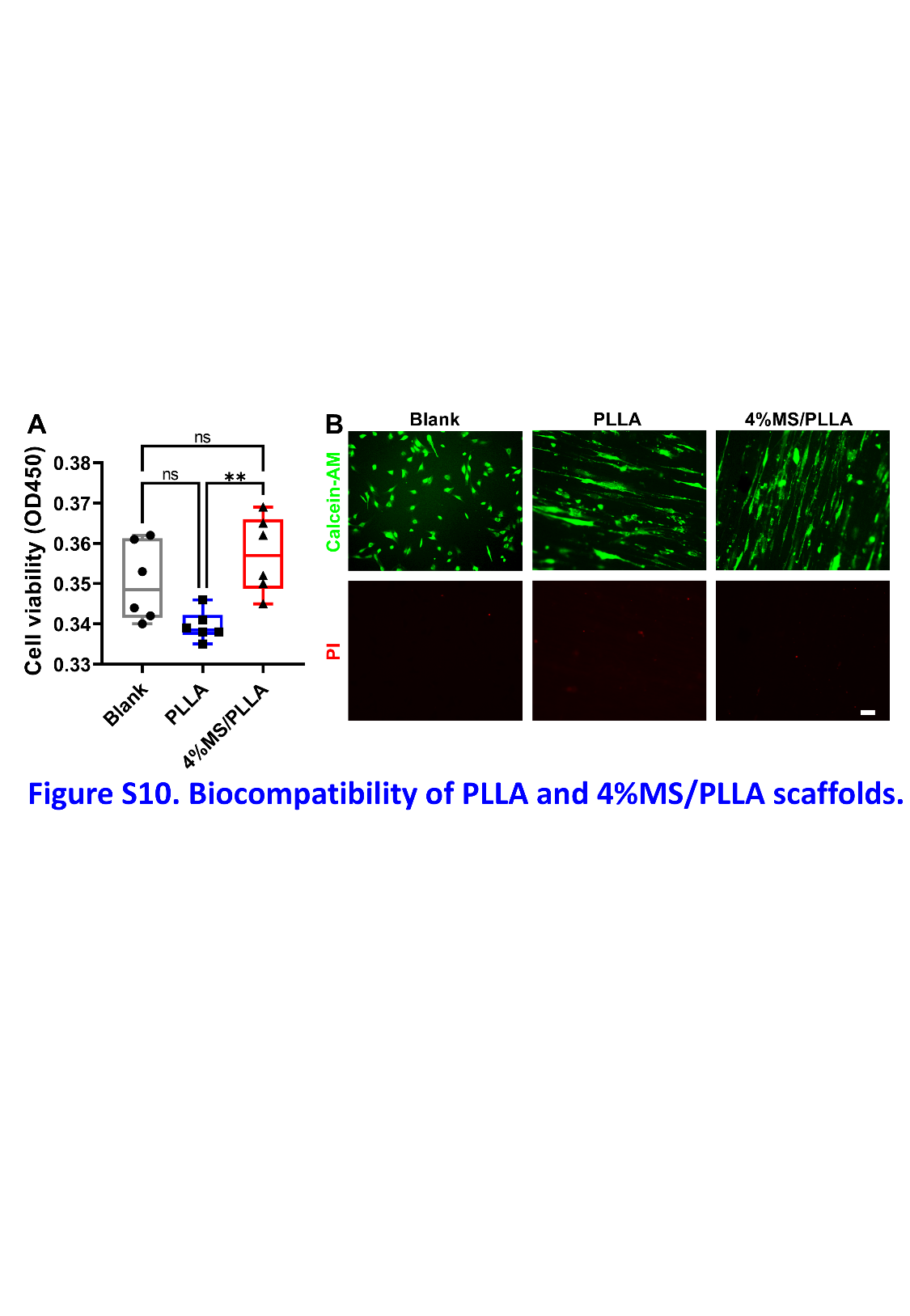


**Figure S10. Biocompatibility of PLLA and 4%MS/PLLA scaffolds.** A. CCK-8 assay to detect the cell viability of MuSCs. B. Live-Dead assay to detect the survival of MuSCs. Green: Calcein-AM, represents live cells; Red: PI, represents dead cells. Scale bar: 100 μm.


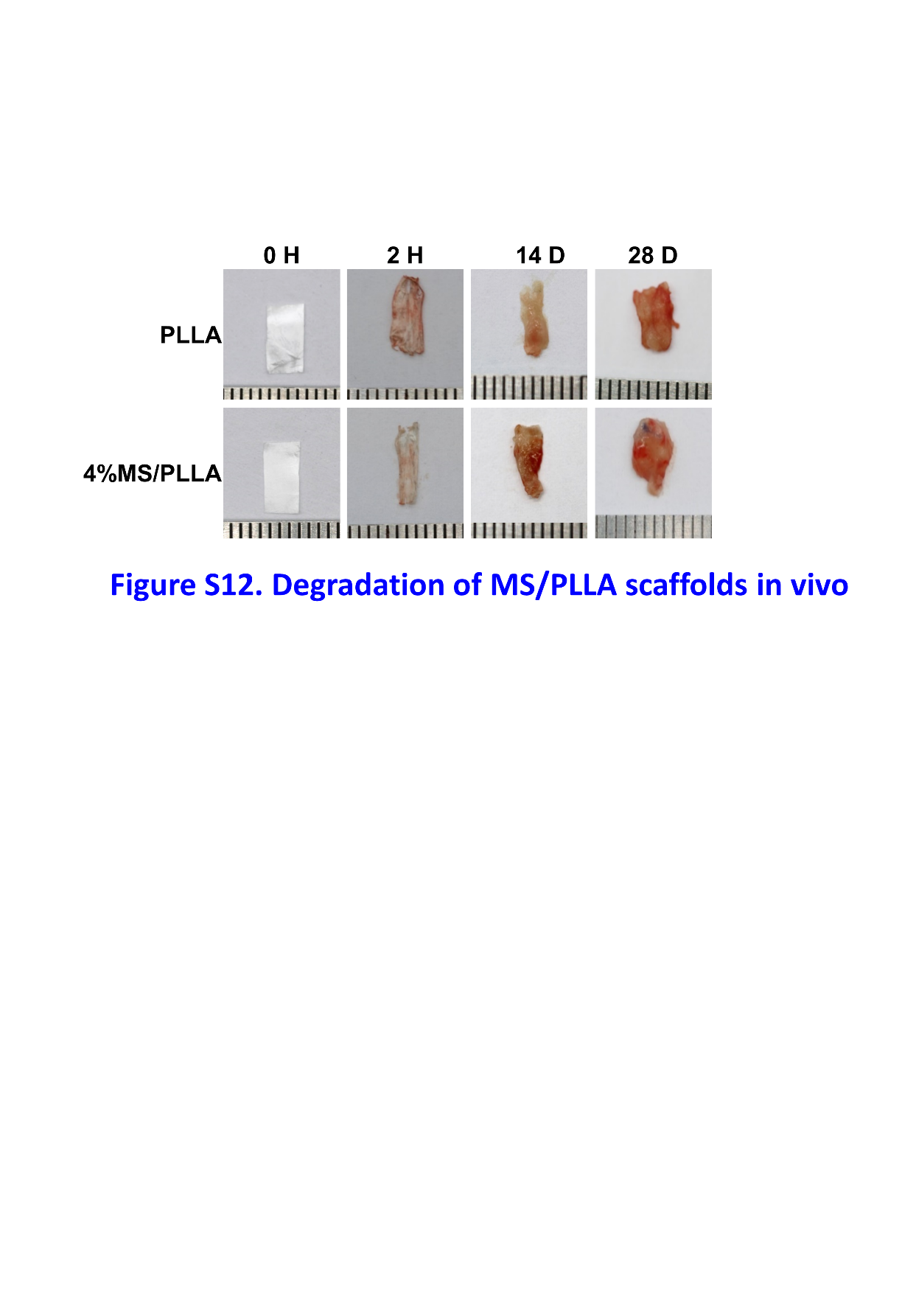


**Figure S11.** **Photographs of the implanted scaffolds at different time points *in vivo.***


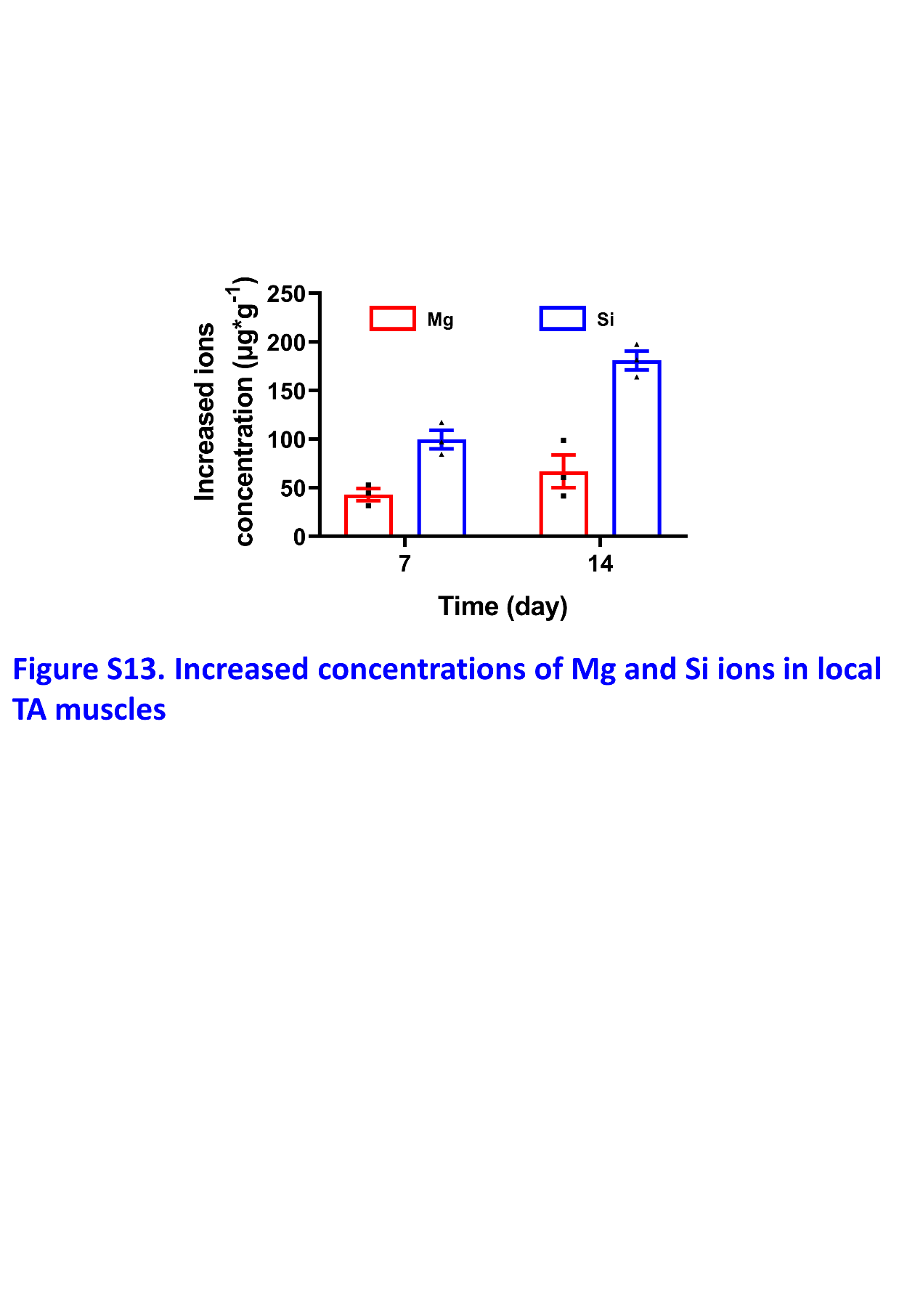


**Figure S12. Increased concentrations of Mg and Si ions in local TA muscles after the implantation of 4%MS/PLLA.**

**
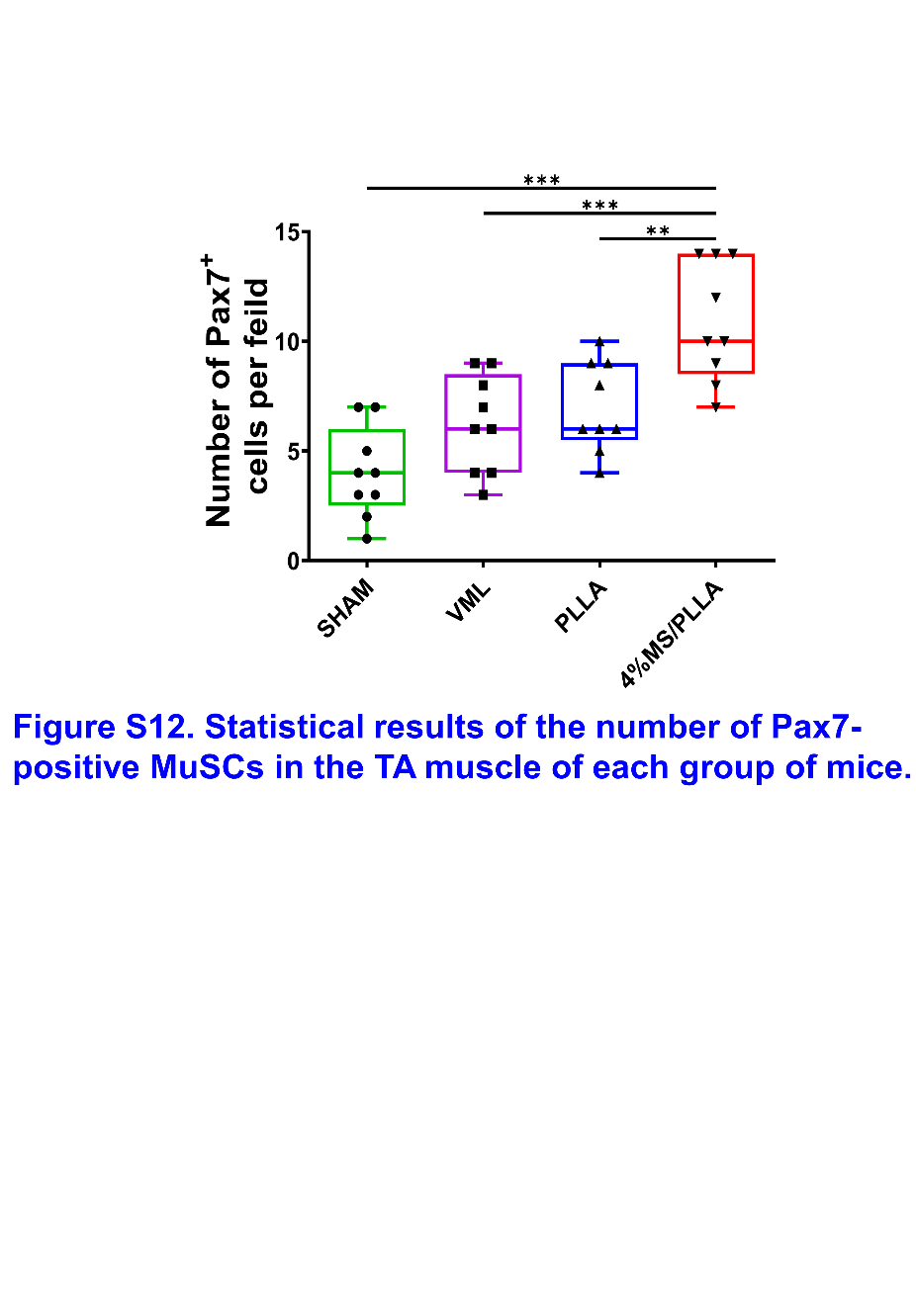
**

**Figure S13. Statistical results of the number of Pax7-positive MuSCs in the TA muscle of each group of mice.** **, p<0.01. ***, p<0.001.

**Supplementary Table S1. Primer sequences used for qRT-PCR**

| Gene Name | Sequences of primers (5’-3’) | |
| --- | --- | --- |
|  | Forwards | Reverse |
| Gapdh | TGGATTTGGACGCATTGGTC | TTTGCACTGGTACGTGTTGAT |
| Myod | ACCCGAAGACTGCTGTGTC | GTCTGACGATTCACAACAGGC |
| Myog | CTGTTTAAGACTCACCCTGAGAC | GGTGCAACCATGCTTCTTCA |
| Mymk | TTCCTCCCGACAGTGAGCAT | GCACAGCACAGACAAACCAG |
| Mymx | TTGTCCTCAGCCAGCAACAG | GGGCCCAATCTCTCCTTCCT |
| Myhc1 | TTGAAAAGACGAAGCAGCGAC | AGAGAGCGGGACTCCTTCTG |
| Notch1 | GATGGCCTCAATGGGTACAAG | TCGTTGTTGTTGATGTCACAGT |
| Hes1 | CCAGCCAGTGTCAACACGA | AATGCCGGGAGCTATCTTTCT |

**Supplementary Table S2. Information of antibodies used in this study**

Primary antibodies used in the study

| Primary antibody | Type | Company | Catalog No. | Dilution rate |
| --- | --- | --- | --- | --- |
| Pax7 | Monoclonal | SANTA CRUZ | sc-81648 | 1:200 |
| Myod | Monoclonal | SANTA CRUZ | sc-377460 | 1:200 |
| Myogenin | Polyclonal | Bioss | bs-3550R | 1:200 |
| Myosin | Monoclonal | BOSTER | BM0096 | 1:200 |
| Laminin | polyclonal | Merck | L9393 | 1:200 |
| CD31 | polyclonal | Servicebio | GB11063-2 | 1:200 |
| Notch1 | polyclonal | Bioss | bs-1335R | 1:200 |
| Hes1 | polyclonal | Bioss | bs-2972R | 1:200 |
| Gapdh | Monoclonal | Beyotime | AF0006 | 1:1000 |

Secondary antibody used in the study

| Secondary Antibody | Conjugate Used | | Company | | Catalog No. | Dilution rate |
| --- | --- | --- | --- | --- | --- | --- |
| Goat Anti-mouse IgG | | HRP | | Cell Signaling Technology | 7076 | 1:3000 |
| Goat Anti-rabbit IgG | | HRP | | Cell Signaling Technology | 7074 | 1:3000 |
| Goat Anti-rabbit IgG | | Cy3 | | Servicebio | GB21303 | 1:100 |
| Goat Anti-rabbit IgG | | FITC | | Servicebio | GB22303 | 1:100 |
| Goat Anti-mouse IgG | | Cy3 | | Servicebio | GB21301 | 1:100 |
| Goat Anti-mouse IgG | | FITC | | Servicebio | GB22301 | 1:100 |
